# NMR-Based Metabolomic Analysis of Spatial Variation in Soft Corals

**DOI:** 10.3390/md12041876

**Published:** 2014-03-28

**Authors:** Qing He, Ruiqi Sun, Huijuan Liu, Zhufeng Geng, Dawei Chen, Yinping Li, Jiao Han, Wenhan Lin, Shushan Du, Zhiwei Deng

**Affiliations:** 1 College of Chemistry, Beijing Normal University, Beijing 100875, China; E-Mails: 163_heqing@mail.bnu.edu.cn (Q.H.); sunruiqi0402@gmail.com (R.S.); Xinjiang20041021@163.com (Y.L.); hanjiao19890516@163.com (J.H.); 2 Beijing Forensic Science Institute, Beijing 100875, China; E-Mail: huijuan0709@163.com; 3 Analytic and Testing Center, Beijing Normal University, Beijing 100875, China; E-Mail: gengzhufeng@bnu.edu.cn; 4 China National Center for Food Safety Risk Assessment, Beijing 100022, China; E-Mail: dila2006@163.com; 5 College of Chemistry and Chemical Engineering, Xinjiang Normal University, Xinjiang 830000, China; 6 State Key Laboratory of Natural and Biomimetic Drugs, Peking University, Beijing 100083, China; E-Mail: whlin@bjmu.edu.cn; 7 State Key Laboratory of Earth Surface Processes and Resource Ecology, Beijing Normal University, Beijing 100875, China; E-Mail: dushushan@bnu.edu.cn

**Keywords:** soft coral, NMR spectroscopy, chemometrics, spatial variation

## Abstract

Soft corals are common marine organisms that inhabit tropical and subtropical oceans. They are shown to be rich source of secondary metabolites with biological activities. In this work, soft corals from two geographical locations were investigated using^ 1^H-NMR spectroscopy coupled with multivariate statistical analysis at the metabolic level. A partial least-squares discriminant analysis showed clear separation among extracts of soft corals grown in Sanya Bay and Weizhou Island. The specific markers that contributed to discrimination between soft corals in two origins belonged to terpenes, sterols and *N*-containing compounds. The satisfied precision of classification obtained indicates this approach using combined ^1^H-NMR and chemometrics is effective to discriminate soft corals collected in different geographical locations. The results revealed that metabolites of soft corals evidently depended on living environmental condition, which would provide valuable information for further relevant coastal marine environment evaluation.

## 1. Introduction

Soft corals refer to the marine colonial organisms in the class Octocorallia that inhabit tropical and subtropical area including South China Sea [1]. As scientists’ concerns about the compounds with interesting medical properties have increased, a number of bioactive secondary metabolites such as sesquiterpenes, diterpenes, sterols and alkaloids produced by soft corals have been found. In the area of drug research, these natural products were proved to possess diverse bioactivities such as anti-inflammation, anti-tumor and antioxidant activity [1]. They were potential candidates for novel medicinal drugs.

Several studies have reported the spatial diversity of chemical composition in soft corals. Koh *et al*.’s research showed different individuals of same species contained different types of major cembranoids, and the result indicated the composition of cembranoids in the *Sarcophyton* genus is related with the location where individual samples collected [2] rather than morphological species. The investigation of metabolites from cultured and wild-type soft coral *Klyxum simplex* resulted in different diterpenoids. Simplexins A–I were isolated from the wild-type soft coral, and new eunicellin-base diterpenoids, klysimplexins and klysimplexin sulfoxides, were obtained from cultured *K. simplex* [3,4,5]. A report on the soft coral *S. flexibilis* showed the metabolites of this sample were abundant in steroids with a small amount of cembranoid diterpenes which were different with previous researches. The authors ascribed the variation to different chemical environment [6].

In this work, we tried to find whether obvious difference exist between soft corals from two geographical origins in the case of various species. Metabolite fingerprinting, coupled with multivariate data analysis, has been applied to give the overview of the metabolic state of biological samples and reveal the changes in measured metabolites due to external perturbations. Environmental metabolomics is one of these applications to study the organism—environment interactions [7]. A wide range of biological systems such as microbes [8,9], plants [10,11], animals [12,13,14,15,16] and other complex biosystems have been investigated to understand the metabololic responses of organisms to environmental stress [17,18]. ^1^H-NMR spectroscopy has been considered to be one of the powerful tools in metabolomic research. The information related to plenty of metabolites could be obtained fast and simultaneously with good reproducibility [19]. Due to the great advantages in information acquisition, NMR-based metabolomics has been used increasingly in marine environment studies. In studies of a marine mussel species, *Mytilus galloprovincialis*, Fasulo *et al*. successfully applied NMR-based metabolomics to assess the metabolic responses of mussels to environment pollution [12]. Healthy California red abalone (*Haliotis rufescens*) and the WS-RLP-infected ones could be well characterized by using the NMR-based metabolomics approach, even in periods of environmentally stress [20]. Impact of environment pollution on caged mussels was investigated using ^1^H-NMR spectroscopy and pattern recognition analysis by transplanting mussels to the contaminated field site. Significant changes of metabolites revealed that metabolomic is an effective tool in assessing environmental influences [13].

In the present work, we investigated the natural variation in metabolites of soft corals from two collection sites in the South China Sea. The metabolic fingerprint and statistical analysis were used to find the metabolites that contribute to the differences between the two groups. In particular, the modeling results from methanol crude extracts and segmented samples were compared. The possible environmental factors for this discrimination were discussed.

## 2. Results and Discussion

### 2.1. Spectral Analysis

**Figure 1:**
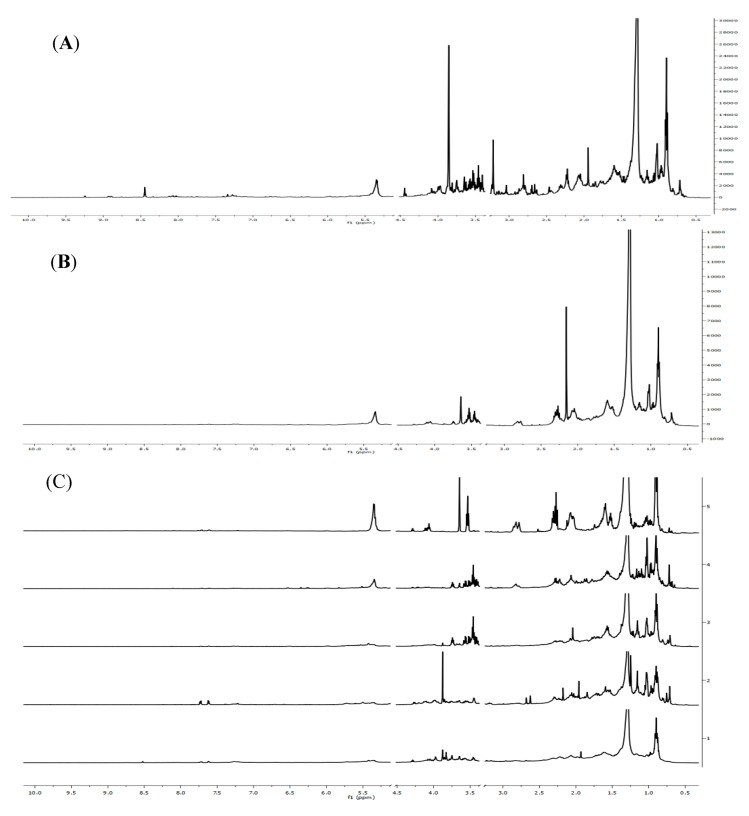
(**A**) The representative ^1^H-NMR spectra of methanol crude extract from *Sinularia capillosa*; (**B**) ^1^H-NMR spectra of ethyl acetate extract; (**C**) Five fractions of ethyl acetate extract.

The representative NMR spectra of methanol crude extract, ethyl acetate extract and Fr.1~Fr.5 from *Sinularia capillosa* collected in Sanya Bay were shown in Figure 1. Figure 1A can be divided into three spectral regions characterized by specific compound resonances. The aliphatic region (0.5–3.0 ppm) is the dominant part (mainly containing fatty acids, terpenes and steroids signals) showing strong signal overlap for all samples. The carbohydrate region (3.0–6.0 ppm) also appears very crowded mainly due to glucoside signals. In contrast, peaks in aromatic region (6.0–10.0 ppm) is sparse with low intensity. 

In our previous studies, ethyl acetate extraction is a suitable method to remove the majority of primary metabolites such as fatty acids and sugars. Bioactive compounds such as terpenes were mainly in ethyl acetate extract [21]. The problem of signal overlap still existed in the spectra of ethyl acetate extract. A further fractionation was applied as a pre-treatment method to simplify the spectra and facilitate assignment [22]. The assignment was accomplished on the NMR spectra of five fractions by comparison to pure compounds. In Table 1, molecular structures of seven assigned metabolites were listed.

Unlike other literature reports of genus *Sinularia*, the systematically chemical analysis result showed the major constituents of terpenes were not the common cembrenes but a number of sesquiterpenoids with various skeleton types. The chemical diversity of the terpenes is depending on collection locations, which has been already observed for other soft corals, *Cladiella krempfi* [23] and *Sinularia flexibilis* [24,25]. 

To provide a comprehensive interpretation of the metabolites differences in soft corals according to two different geographic origins, statistical analysis was applied to ^1^H-NMR spectral data sets. 

### 2.2. Statistical Analysis

Initially, crude extracts data and fractions data were subjected to PCA respectively and the outliers lying outside of 95% confidence limits on PC scores were removed to ensure the data reliability. PCA analysis did not well group samples between different geographical locations.

Subsequently, statistical analysis PLS-DA was performed on ^1^H-NMR spectra of methanol crude extracts. A classification model was established for discrimination between geographic origins. The optimal number of latent variables (LVs) determined by cross-validation (CV) was six. These latent variables adequately captured 91.6% of total variance with R^2^Y = 94.0% and Q^2^ = 32.7%. In addition, a permutation test was applied to check the validity of PLS-DA model. The result showed that it is quite likely to obtain lower goodness of fit (R^2^) and predictive ability (Q^2^) when the observations are permuted at random.

**Figure 2:**
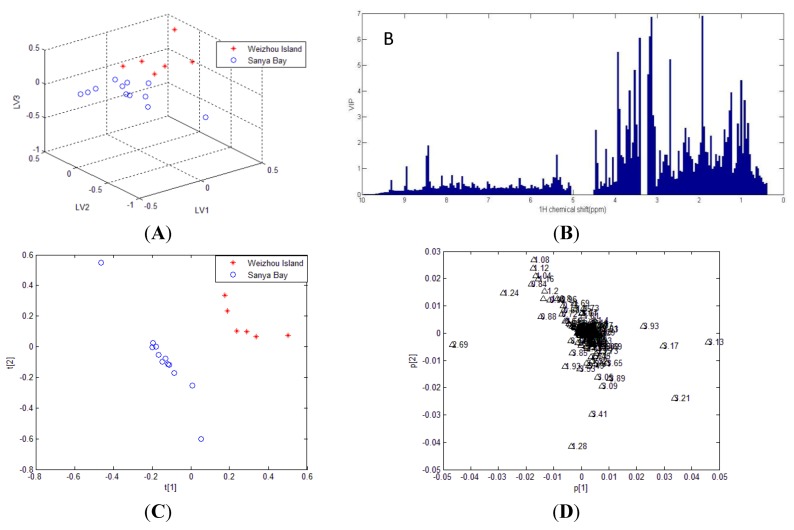
Partial least squares discriminant analysis (PLS-DA) and OPLS-DA results of ^1^H-NMR data of methanol crude extracts from Weizhou Island (red asterisk) and Sanya Bay (blue circle). (**A**) PLS-DA scores plot and (**B**) corresponding VIP plot; (**C**) OPLS-DA scores plot; (**D**) OPLS-DA loading plot.

The PLS-DA scores plot of first three LVs was shown in Figure 2. A satisfied separation among methanol crude extracts was obtained based on origins. This result reveals the metabolites difference of soft coral origins is significant in spite of different species. 

**Table 1:** ^1^H chemical shifts (ppm) and coupling constants (Hz) of *Sinularia capillosa* metabolites identified on the basis of 1D and 2D experiments of pure compounds isolated from ethyl acetate extract.

Metabolite	Structure	^1^H Chemical Shifts (ppm) and Coupling Constants (Hz)
∆^9(15)^-africanene [26]	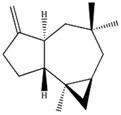	0.23 (dd, *J* = 4.0, 5.0)
d-aromadendrane-4β,10α-diol [27,28]	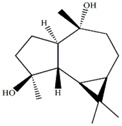	0.34 (t, *J* = 10.5)
(+)-aromadendrane-4α,10β-diol [27,28]	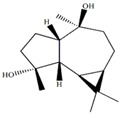	0.01 (t, *J* = 9.5)
Alismoxide [29]	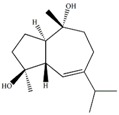	5.46 (s)
dendronpholide O [21]	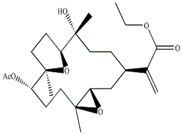	6.24 (s), 5.76 (s)
4(15)-eudesmene-1β,6α-diol [30,31]	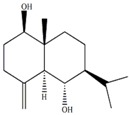	4.85 (s), 4.72 (s)
Germacra-4(15),5,10(14)-trien-1α-ol [32]	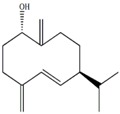	5.82 (d, *J* = 16.2), 4.96 (s), 3.85 (m)

The VIP (Variable Importance in the Projection) scores were calculated to investigate the highly influential variables of the given PLS model. Since the average of all X variables scores equals to one, these variables with largest scores (VIP > 1) were simply selected as the most affecting ones [33]. It was evident that significant contribution to group separation was concentrated in carbohydrate region as shown in Figure 2B. The OPLS-DA model was established to exclude variations that are not correlated to Y matrices. Figure 2D is a coefficient plot showing biomarkers with high contributions to differentiation. Some glucosides Signals, such as the resonances at δ_H_ 3.13, 3.17, 3.21 and 3.93, were evident higher in Weizhou Island group compared with Sanya Bay group. The concentration of lipid (identified by its characteristic resonance at δ_H_ 1.28) was lager in soft corals from Sanya Bay.

**Figure 3:**
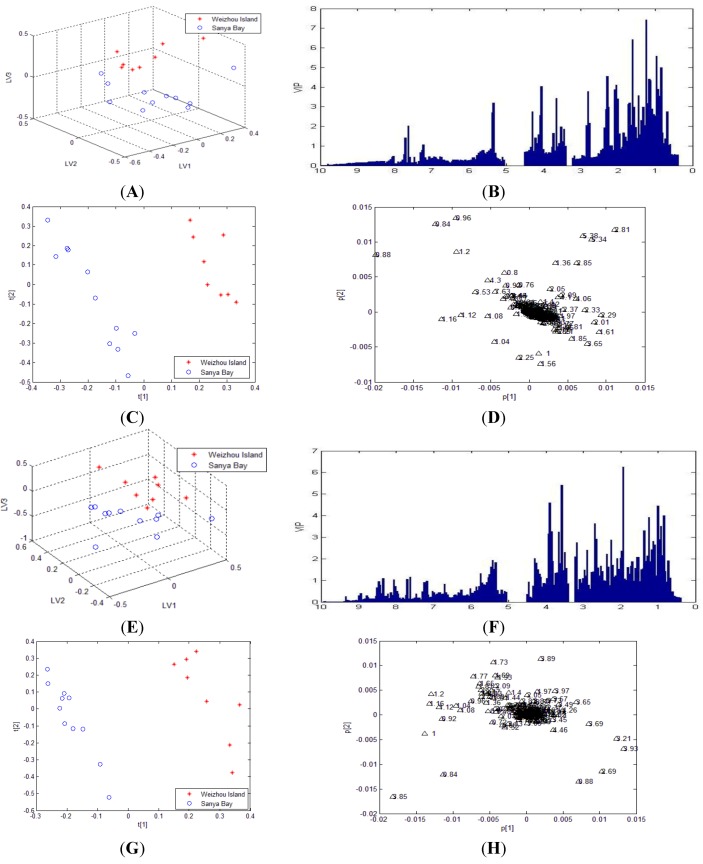
PLS-DA and OPLS-DA results of ^1^H-NMR data of Fr.1 and Fr.5 from Weizhou Island (red asterisk) and Sanya Bay (blue circle). (**A**) PLS-DA scores plot for Fr.1; (**B**) PLS-DA VIP plot showing the major variations for Fr.1; (**C**) OPLS-DA scores plot for Fr.1; (**D**) OPLS-DA loading plot for Fr.1; (**E**) PLS-DA scores plot for Fr.5; (**F**) PLS-DA VIP plot for Fr.5; (**G**) OPLS-DA scores plot for Fr.5; (**H**) OPLS-DA loading plot for Fr.5.

However, since the signal intensity in aromatic region was too low to be effective in the selected calculation model under the existence of large amount of primary metabolite signals, the previous PLS-DA analysis based on methanol crude extracts did not show us the variation of these minor second metabolites which we are concerned about between different groups. In order to decipher the intensity variation of weak peaks of second metabolites between Sanya Bay and Weizhou Island samples, PLS-DA was applied to NMR data obtained from all segmented samples. Calculated results for PLS-DA models were listed in Table 2. It was shown that the PLS-DA model created on Fr.1 showed better robustness and predictability than that on methanol crude extracts. However, the PLS-DA model created on Fr.2~Fr.4 showed poor predictive ability according to the value of cross-validated Q^2^. From the results, it might be assumed that less information related to classification was contained in these segmented spectra. Permutation tests were performed on all models. The results indicated better predictive power of Fr.1 and Fr.5 model compared to methanol extract model. The clear separation observed in Figure 3A, E proved further fractionation using gradient solvents mixture an effective method in metabolomic characterization of soft coral samples.

**Table 2:** Component vector values and cross-validated Q^2^ of leave-one-out test for PLS-DA models.

PLS-DA Model Data	R^2^X	R^2^Y	Cross-validated Q^2^
^1^H-NMR data of methanol extract	0.916	0.940	0.327
^1^H-NMR data of ethyl acetate extract Fr.1	0.977	0.971	0.610
^1^H-NMR data of ethyl acetate extract Fr.2	0.747	0.505	0.207
^1^H-NMR data of ethyl acetate extract Fr.3	0.968	0.790	0.176
^1^H-NMR data of ethyl acetate extract Fr.4	0.846	0.845	0.175
^1^H-NMR data of ethyl acetate extract Fr.5	0.815	0.573	0.371

The maker signals of fraction models were also determined by the rank of chemical shift values in VIP selection method. Combined with the corresponding VIP values, the major variation were concentrated in aliphatic region and carbohydrate region for Fr.1 and Fr.5. In order to figure out the major cause of the variation, the literatures were researched about chemical constituents of soft corals obtained from Sanya Bay and Weizhou Island of South China sea. Sterols and steroidal glycosides were generally regarded as two main types of metabolites isolated from soft coral in Weizhou Island. However, terpenes and sterols can be obtained from most soft corals in Sanya Bay. It was shown that the possibility of finding terpenes in Sanya Bay was higher than in Weizhou Island. 

The typical cases reported covers eleven samples from three common soft coral genus (*Sinularia* sp., *Sarcophyton* sp. and *Dendronephthya* sp.). Among them, six (*Sinularia* sp., *n* = 3; *Sarcophyton* sp., *n* = 2; *Dendronephthya* sp., *n* = 1) were from Sanya Bay and five (*Sinularia* sp., *n* = 3; *Sarcophyton* sp., *n* = 1; *Dendronephthya* sp., *n* = 1) from Weizhou Island. It is worth noting that although *Sinularia* sp. have been found to be a rich source of terpenes especially cembrenes [34,35,36,37], no individuals from Weizhou Island were found containing terpenes as major constituents of secondary metabolites [38,39,40]. This situation was also observed in *Sarcophyton* sp. The soft coral of Sanya Bay provides a series of cembranoids and tetraterpenoids which were not found in sample from Weizhou Island [41,42,43]. Studies on chemical examination of the genus *Dendronephthya* resulted in the polyhydroxysteroids as main metabolites. Lin *et al*. reported an isolation of 18 new cembrane-type diterpenes, 11-episinulariolide and sandensolide from *Dendronephthya* sp. collected in Sanya Bay [21]. 

To further demonstrate the differences between two groups, OPLS-DA was employed to give an improved discrimination and help identifying potential significant metabolites. Before statistical analysis, lipid signal (large peak of -(CH_2_)_n_- in 1.24–1.32 ppm region) was removed from raw data of Fr.1 and Fr.5 to reveal the contribution of other biomarkers. The OPLS-DA models demonstrated better separation between different sample groups. From the loading plots, increased signal intensity at the region of 1.0–2.25 ppm in soft corals from Sanya Bay was in accordance with our investigation since signals of terpenes were concentrated in this area. In addition to this region, resonances intensity at δ_H_ 2.81, 2.85, 5.34 and 5.38 were higher in samples from Weizhou Island. The resonances at 5.34 and 5.38 ppm may due to allylic protons of common ∆5-Sterols [44].

Therefore, the significant difference was possibly due to increased levels of sterols and steroidal glycosides, along with decreased levels of terpenes in Weizhou Island samples compared with those in Sanya Bay samples. Sterols and sterol glucosides serve as the precursor for synthesis of steroidal hormones [44] and the significant constituent for maintenance of cell function. Therefore, changes of concentration level could provide information to assess status of soft corals. Based on our investigation, Sanya area represents environmental stress of overfishing (and destructive fishing), high level of terpenes could be presumed as an metabolic response to this anthropogenic disturbance.

## 3. Experimental Section

### 3.1. General Site Description

**Figure 4:**
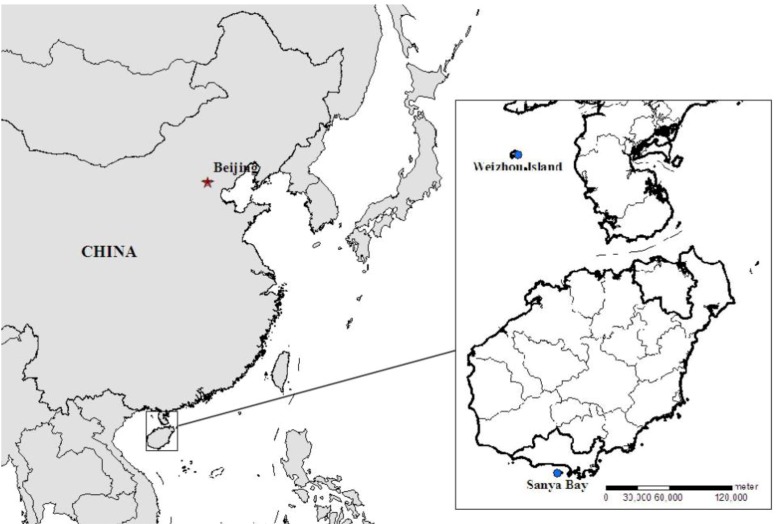
Location map of the studied area.

20 soft coral samples were collected in the area of Sanya Bay (South China sea, China, 18°13′ N, 109°23′ E) and Weizhou Island (South China sea, China, 21°02′ N, 109°09′ E) at the depth of 10 m in the summer season (Figure 4). The physico-chemical parameters of both sites were presented in Table 3. The Sanya Coral Reef National Marine Nature Reserve was established in 1990 and possesses a high diversity of marine habitats in 20th century [45]. The mean value of water temperature is 29.2 °C during the rainy season [46]. Both of the sampling sites are far from coastline and the salinity remains stable around 32 PSU. The level of heavy metals in sediment conforms to National Seawater Quality Standards for China according to The Marine Environment Bulletin of Hainan province published by the government of Hainan Province in each year. According to the investigation of the biodiversity on coastal areas in South China Sea in 2006, the average coral cover was 15.87% in Sanya Bay. The low density and size of fishes in this area indicated the coral reef ecosystems were under severe anthropogenic stress like overfishing (and destructive fishing) [47]. As a threat to Scleractinian corals, outbreaks of *Acanthaster planci* and *Drupella* sp. have been found in adjacent area especially in Yalong Bay during the past few years. 

**Table 3:** Physico-chemical parameters of Sanya Bay and Weizhou Island environment.

Sampling Area	Sanya Bay	Weizhou Island
Temperature(°C)	29.2 [46]	29.6 [48]
Salinity (‰)	33.3 [46]	32.9 [49]
Dissolved oxygen (mg/L)	6.72 [50]	7.31 [48]
pH	8.14 [51]	8.19 [49]
Dissolved Inorganic Nitrogen (mg/L)	0.036 [50]	0.05 [52]
Dissolved Inorganic Phosphate(mg/L)	0.007 [50]	0.0039 [53]

Long-term (from 2005 to 2010) investigation showed the water-related environmental quality of Weizhou Island is well-kept. The concentration of metals in seawater around Weizhou Island was studied in 2011. The results indicate the ecological environment of this region was not polluted (Cu 3.57, Pb 0.35, Zn 6.97, Cr 1.44, Hg 0.017, As 0.44, Cd 0.014 μg/L of water sample) [49]. Although the coral reefs are also faced the problem of anthropogenic stress, the results of reef check in continuous years showed the status of coral reefs is healthy.

### 3.2. Biological Material

Soft coral samples were frozen immediately after collection. Among them, 12 samples were collected from Sanya Bay in Hainan province and 8 samples were collected from Weizhou Island in Guangxi province, respectively. The specimens were identified by Lee P. Van Ofwengen (National Museum of National History Naturalis, The Netherlands) (see Supplementary Table S1 for details), and voucher specimens were deposited at the same museum and also at State Key Laboratory of Natural and Biomimetic Drugs of Peking University, Beijing, China. 

### 3.3. Extraction and Isolation

Methanol extracts: the soft coral samples were homogenized in 95% (v/v) ethanol under room temperature for four times and the concentrated extracts were desalted by dissolving in methanol, then sedimented by 12 h. After filtration, the solutions were concentrated *in vacuo* using a rotary evaporator at 40 °C to give dark brown residuals. 

Ethyl acetate extracts: for each sample, a portion of residuals were redissolved in water and treated with ethyl acetate to obtain the fractions by using two-phase extraction method. This simple fractionation step divided the constituents of methanol crude extract into two parts according to different chemical polarity. The bioactive compounds like terpenes were proved mainly in ethyl acetate extract by applying thin layer chromatography (TLC) and ^1^H-NMR analysis. 

Segmented samples: each of ethyl acetate residues was further chromatographed on a flash column (silica gel), gradient eluting with equal volume of petroleum ether-acetone (from 40:1 to acetone) to give five fractions, which were labeled as Fr.1, 2, …, 5 respectively.

Among these samples, the ethyl acetate extract of soft coral *Sinularia capilosa* was subjected to repeated silica gel column, and purified by preparative HPLC to yield pure compounds by Dawei Chen (Peking University, Beijing, China).

### 3.4. ^1^H-NMR Data Acquisition and Multivariate Analysis

About 10 mg of soft coral samples were dissolved into 500 μL of methanol (d4) containing internal standard as NMR samples. The deuterated solvent methanol (d4) was used to prepare all samples including methanol extracts, ethyl acetate extracts and five fractions for NMR analysis. All spectra were acquired on Bruker DRX 500 spectrometer operating at 500.13 MHz proton frequency using a 5 mm dual probe at 298 K. Solvent suppression was achieved by applying a presaturation scheme with 1D-NOESY presaturation. 256 transients were recorded, as 32 k data points with a spectral width of 8012.82 Hz and a relaxation delay of 5.0 s. A line broadening function of 0.3 Hz was applied to the FID prior to Fourier transformation. Some secondary metabolites of the representative sample were identified by comparing to the literature and pure compounds isolated from the soft coral *Sinularia capillosa*.

All NMR spectra were phased and baseline corrected with MestReNova software (version 8.1.2; Mestrelab Research SL, Santiago de Compostela, Spain). The solvent region of δ 3.25–3.37 ppm (methanol) and δ 5.12–4.52 ppm (water) were excluded from the spectra, and the residual spectral regions were divided into 0.04-ppm bins over the range of δ 0.4–10 ppm. The data were normalized to the total spectral region and exported in ASCII format containing 222 variables. The data files were imported into MATLAB (R2010a; Mathworks, Inc., Natick, MA, USA). Principal component analysis (PCA) was applied to give a overview of the data distribution. Supervised partial least squares discriminant analysis (PLS-DA and OPLS-DA) models were constructed in order to discriminate soft corals according to their geographic distribution. The PCA and PLS (OPLS) methods were performed by applying PLS Toolbox (Eigenvector Research, Inc., Manson, WA, USA). All the models were cross-validated using a leave-one-out method. The vadility was checked by performing permutation test. The optimal number of latent variables was selected by cross-validation and the performance of PLS model was evaluated by R^2^ (captured variance, “ability to fit the data”) and Q^2^ (cross-validated coefficient, “ability to predict”). The VIP (variable importance in the projection) scores were calculated in order to explain the importance of each variable in a given PLS model. The interesting metabolites were identified by employing loading plots from OPLS models.

## 4. Conclusions

Due to high variability in the composition of secondary metabolites, no systematic studies on patterns of spatial variation in soft corals have been reported. The present work investigated the feasibility of applying ^1^H-NMR fingerprints in combination with supervised partial least squares discriminant analysis to give the variation of soft coral metabolites from different sites. Moreover, by employing further fractionation of ethyl acetate extracts, the PLS-DA and OPLS-DA models showed better prediction abilities. The results obtained on segmented samples shown that the important compounds related to group separation were mostly second metabolites such as terpenes, sterols and glucosides. These metabolites variation could be considered as the response of biosystem to different environment. Since coral reefs are highly sensitive ecosystems, they have been suggested as potential indicators of marine environment [54]. Based on our investigation, the anthropogenic activities near Sanya Bay were considered as the main factor that contributes to the status of coral reefs from Sanya area. As part of this ecosystem, soft corals also faced the problem and the metabolic changes of soft corals could be predicted as a response to environmental stress. Metabolomics may provide valuable information to reveal environment-depended complex acting mechanism of soft corals.

## Supplementary Files

Supplementary File 1 Supplementary Information (PDF, 897 KB)
