# Supplementary material for: NMR-Based Metabolomic Analysis of Spatial Variation in Soft Corals
Source: Mar Drugs. 2014 Mar 28;12(4):1876–90. doi: 10.3390/md12041876 (PMC4012446; doi:10.3390/md12041876)
Supplement: Supplementary File 1 — Supplementary Information (PDF, 897 KB) [file marinedrugs-12-01876-s001.pdf]

Supplementary Information

Table S1. The morphological species and collected location of soft coral samples.

Morphological Species	Collected Location
Dendronephthya sp.	Weizhou Island
Dendronephthya sp.	Weizhou Island
Sinularia sp.	Weizhou Island
Sinularia sp.	Weizhou Island
Cladiella sp.	Weizhou Island
Sarcophyton ehrenbergi	Weizhou Island
unidentified	Weizhou Island
unidentified	Weizhou Island
Sinularia capillosa	Sanya Bay
Sinularia capillosa	Sanya Bay
Sinularia gibberosa	Sanya Bay
Sarcophyton trocheliophorum	Sanya Bay
Dendronephthya sp.	Sanya Bay
unidentified	Sanya Bay
unidentified	Sanya Bay
unidentified	Sanya Bay
unidentified	Sanya Bay
unidentified	Sanya Bay
unidentified	Sanya Bay
unidentified	Sanya Bay

Figure S1. ^{1}H-NMR spectrum for compound **1**.

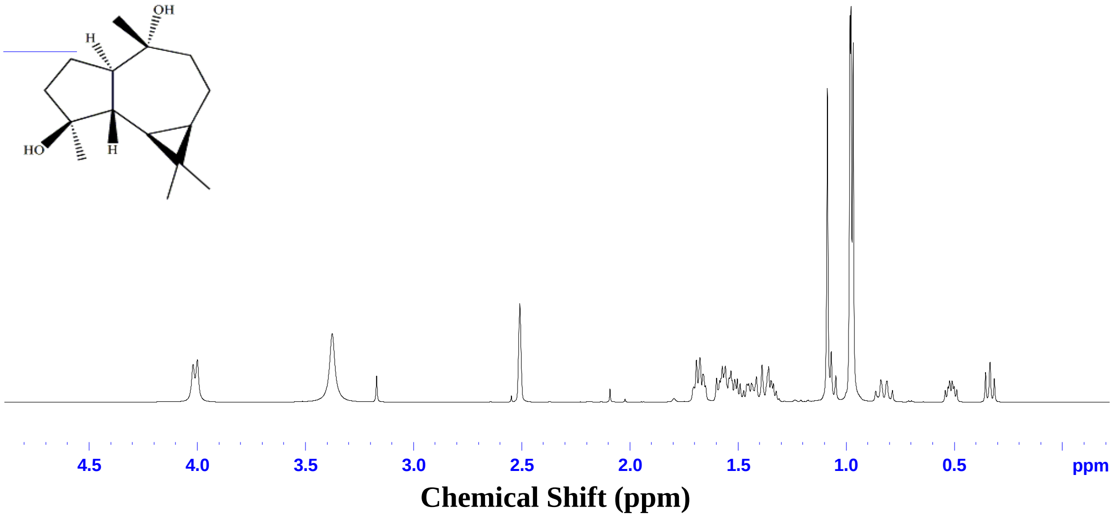

Figure S2. DEPTQ NMR spectrum for compound **1**.

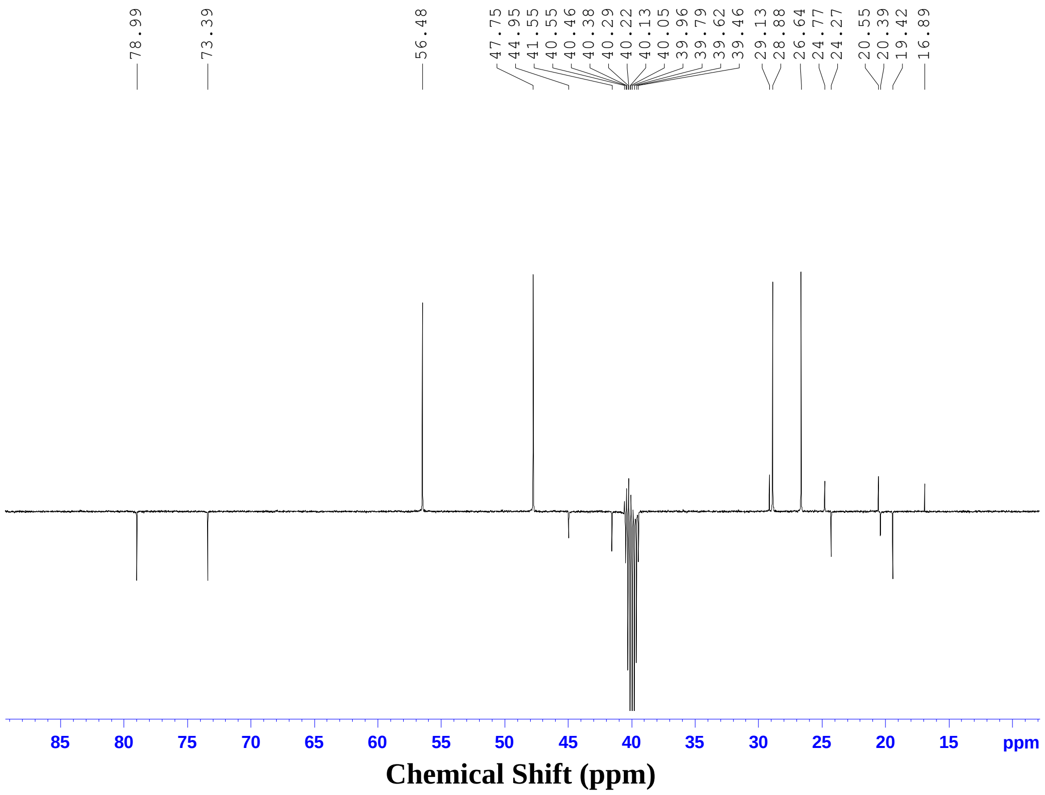

Figure S3. COSY NMR spectrum for compound **1**.

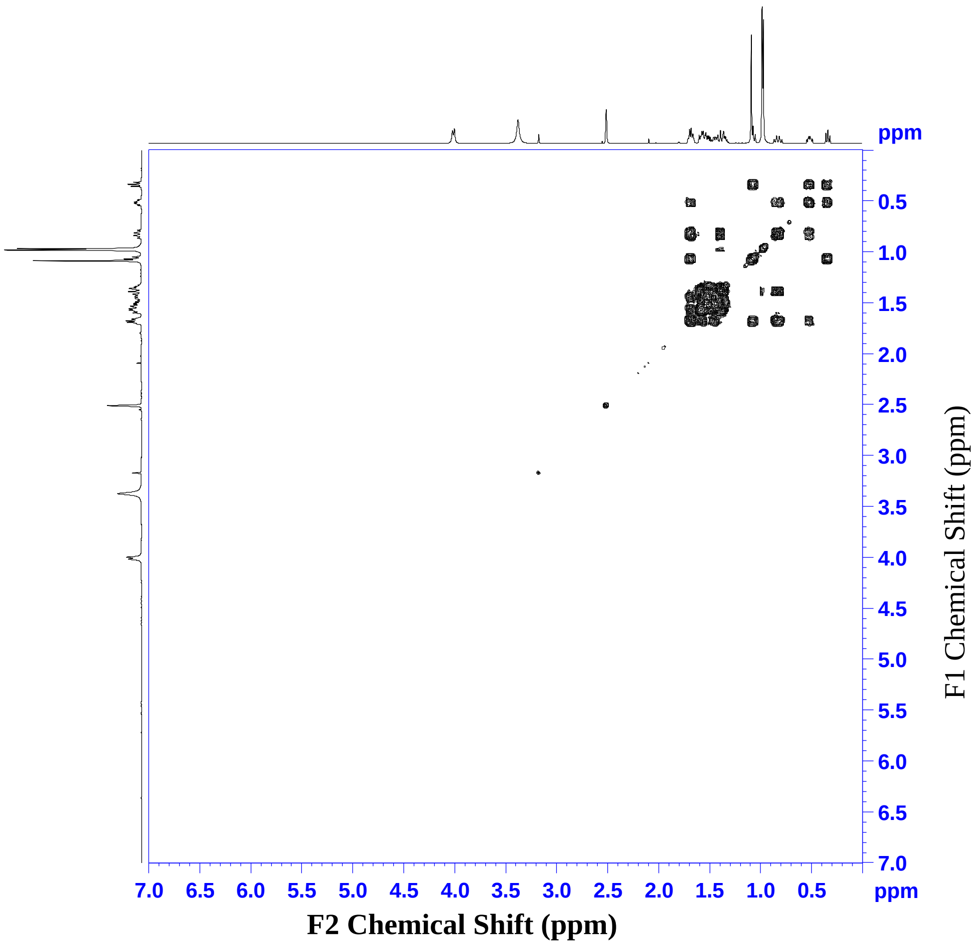

Figure S4. HSQC NMR spectrum for compound **1**.

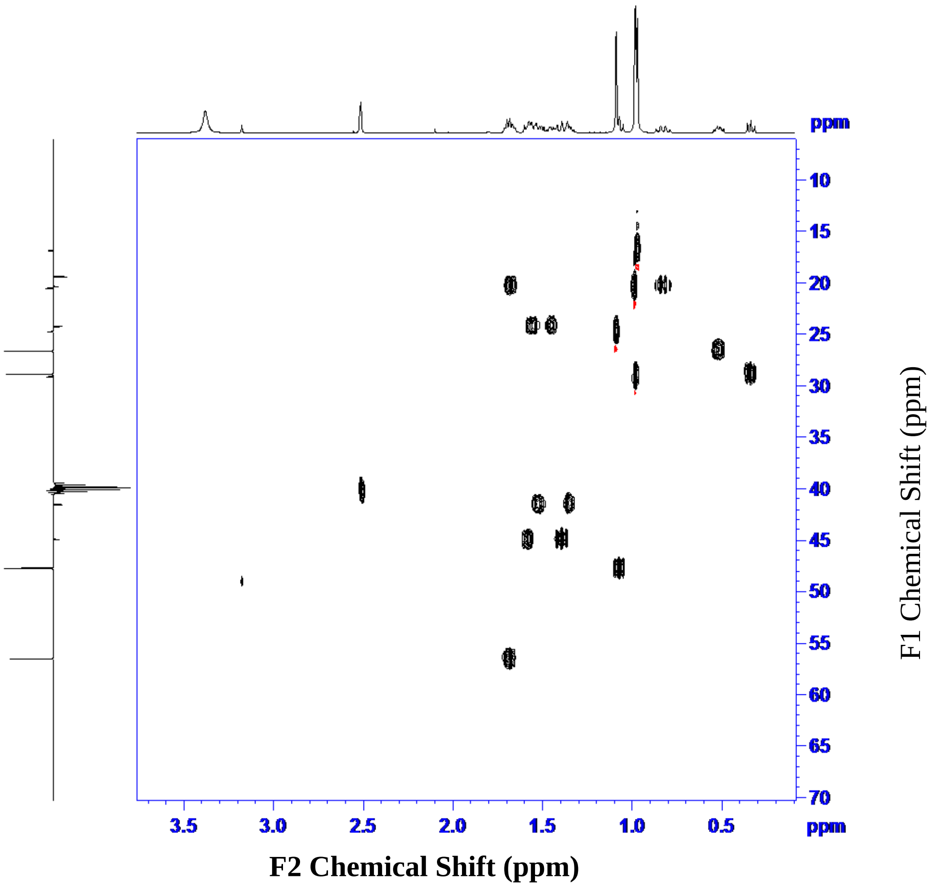

Figure S5. HMBC NMR spectrum for compound **1**.

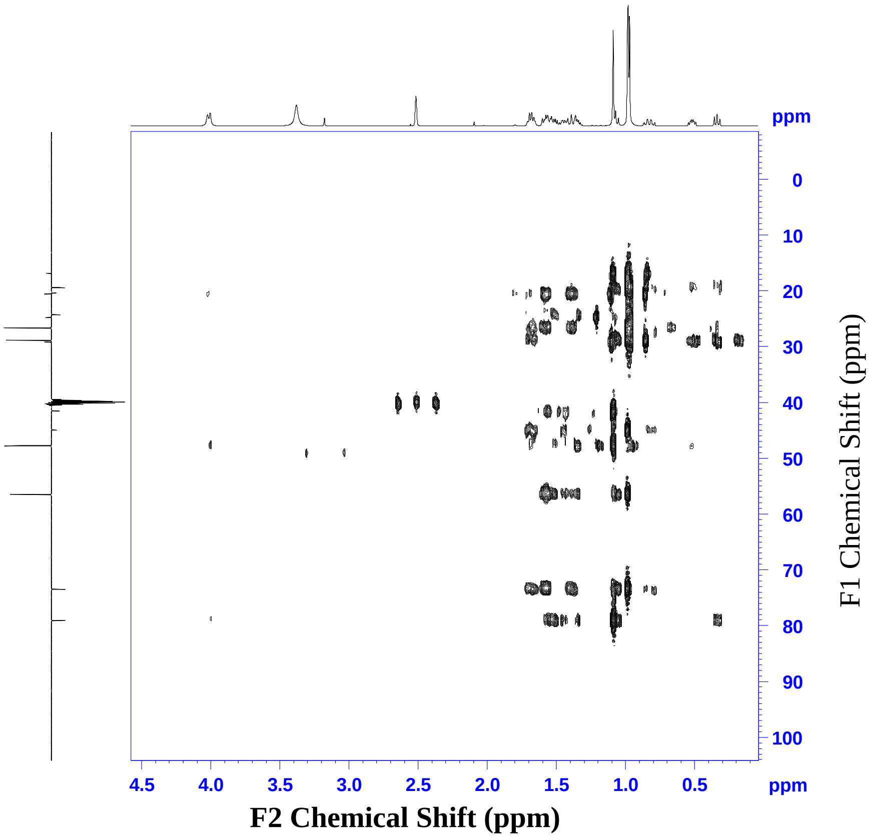

Figure S6. ROESY NMR spectrum for compound **1**.

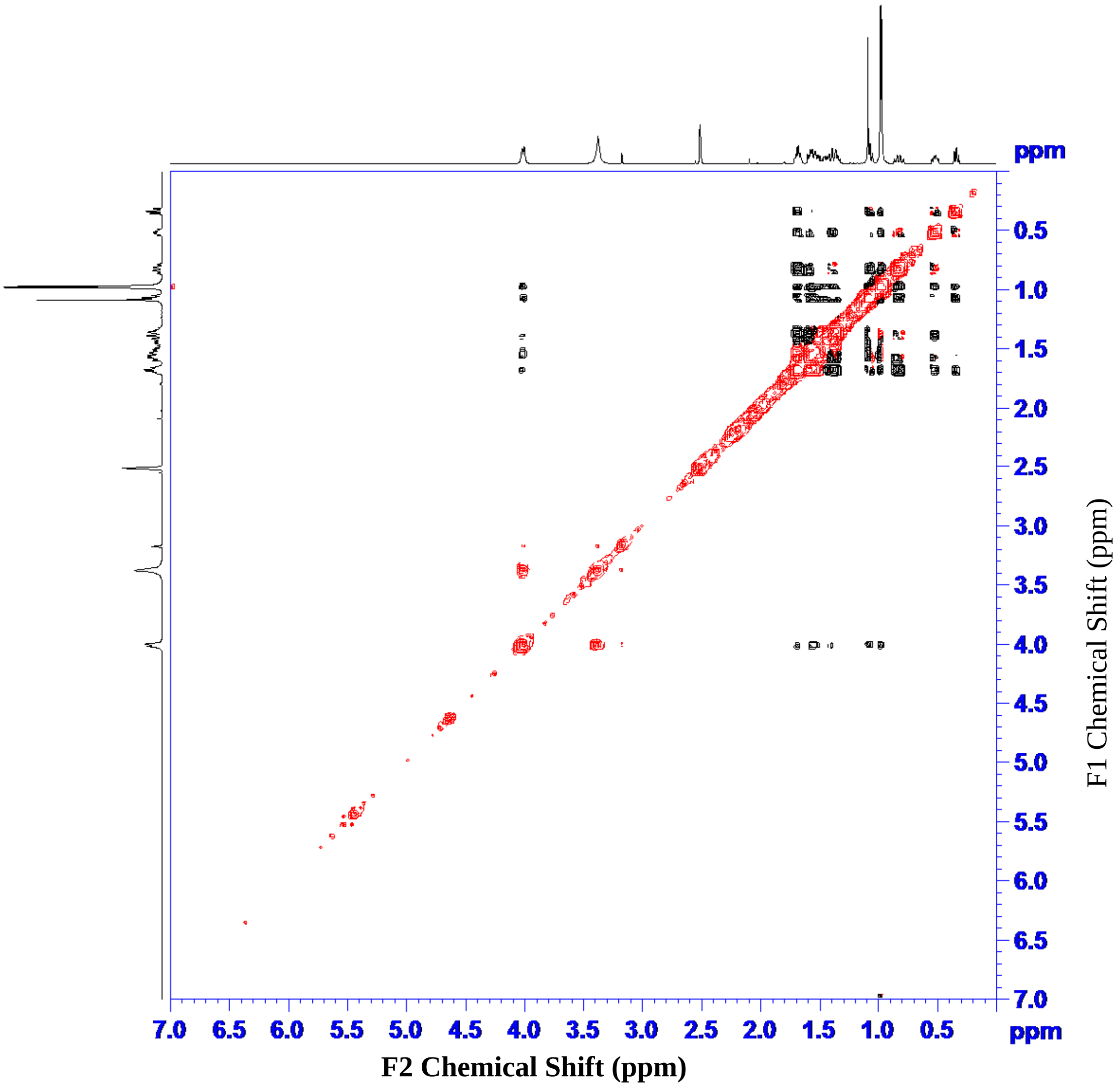

Figure S7. ^{1}H-NMR spectrum for compound **2**.

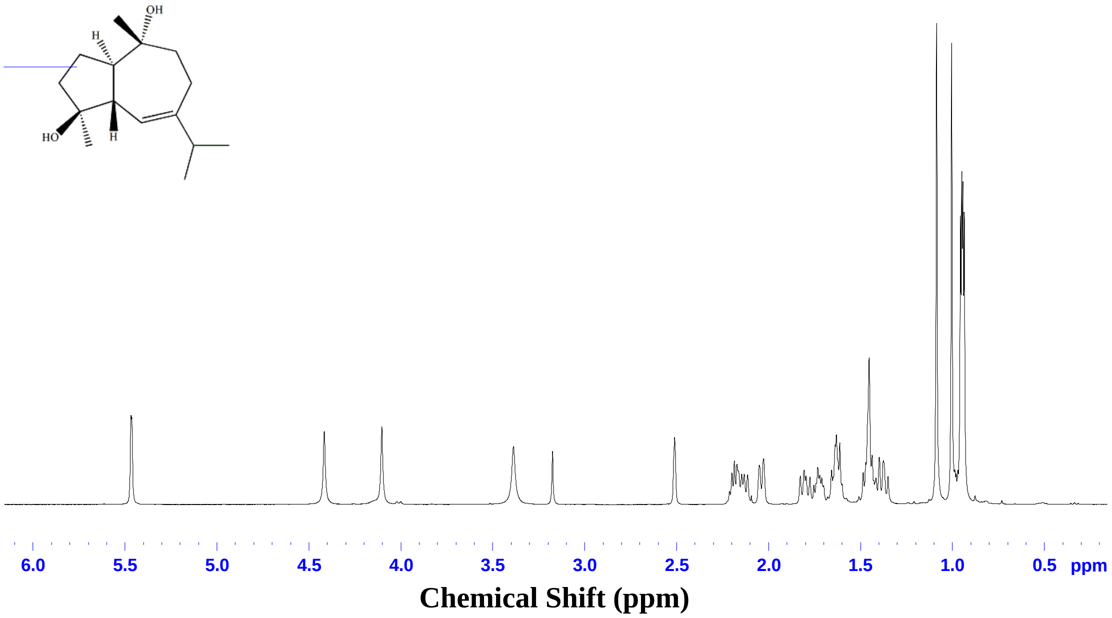

Figure S8. APT NMR spectrum for compound **2**.

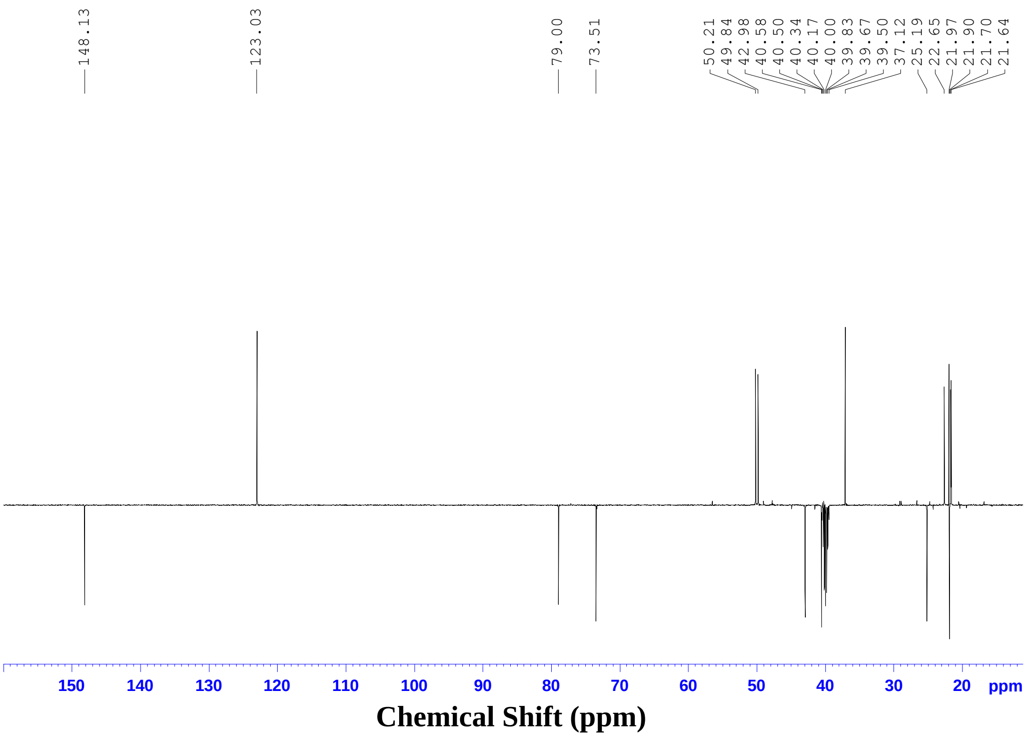

Figure S9. COSY NMR spectrum for compound **2**.

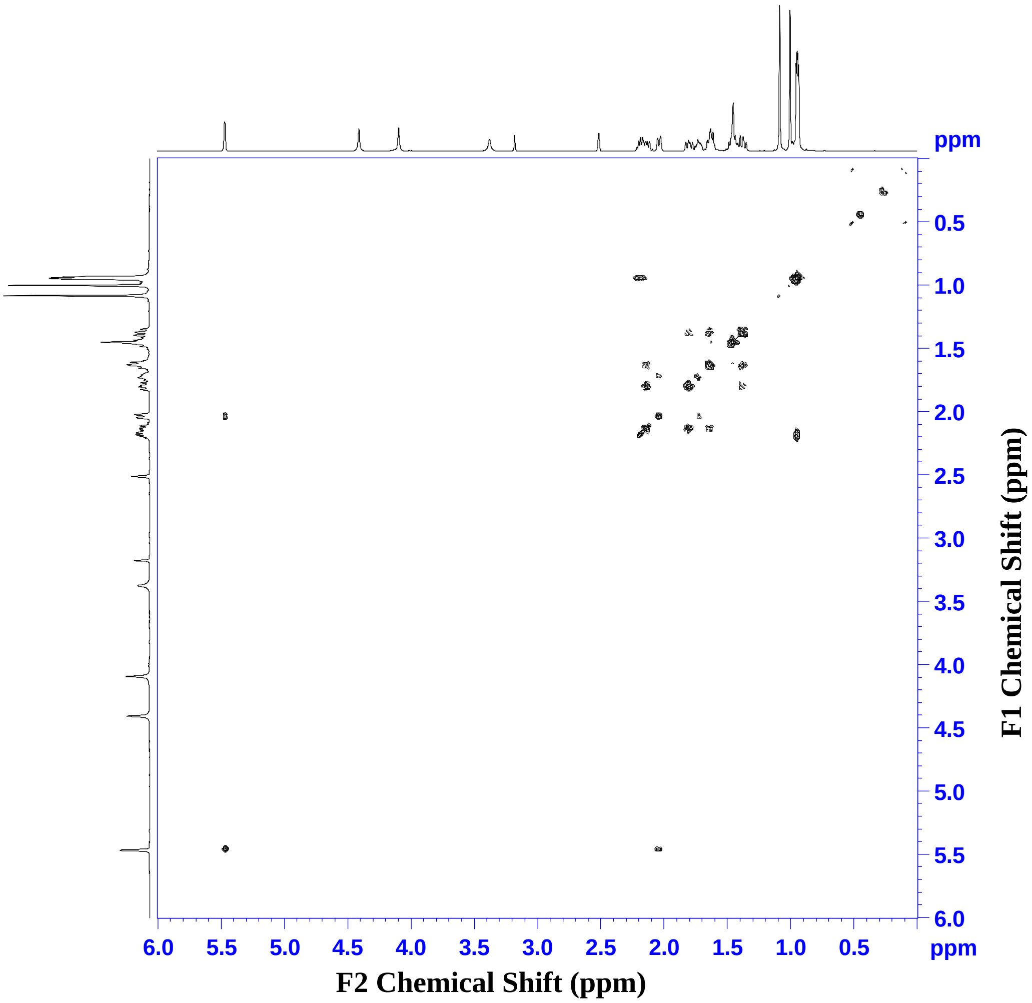

Figure S10. HSQC NMR spectrum for compound **2**.

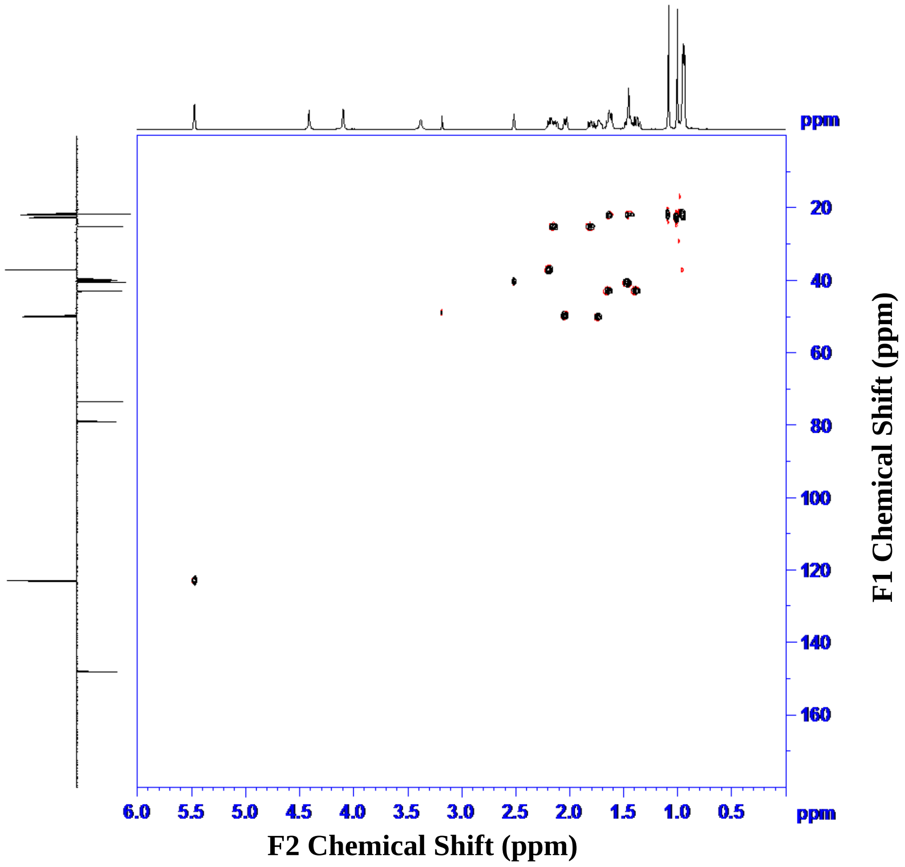

Figure S11. HMBC NMR spectrum for compound **2**.

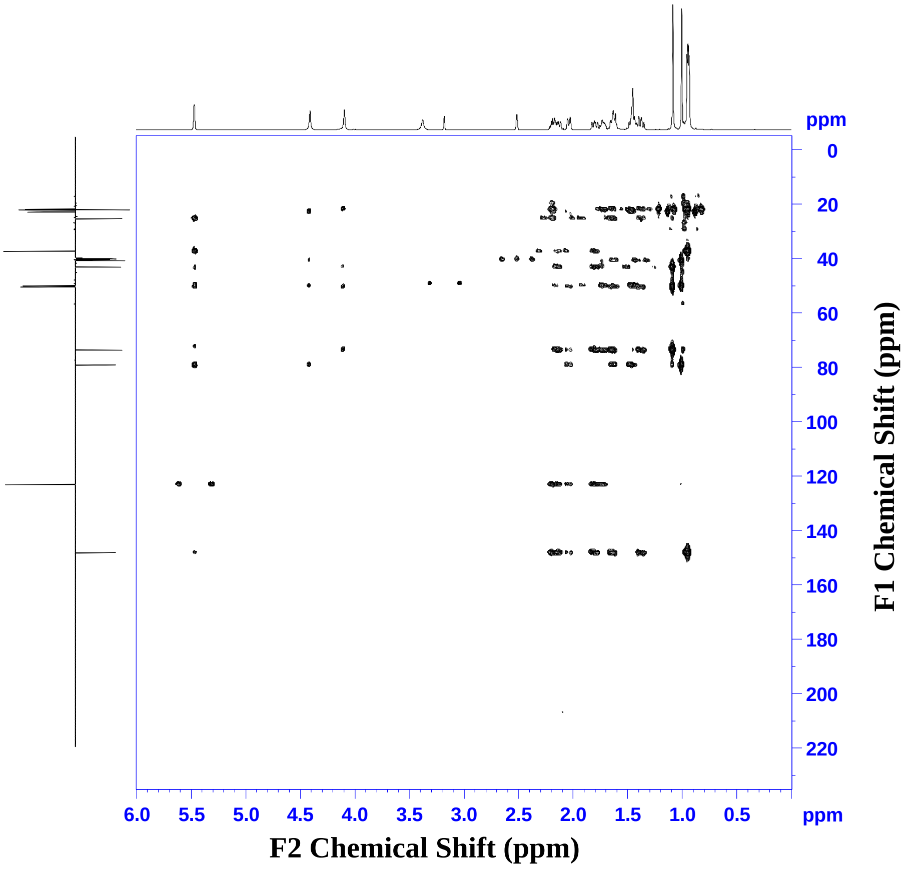

Figure S12. ROESY NMR spectrum for compound **2**.

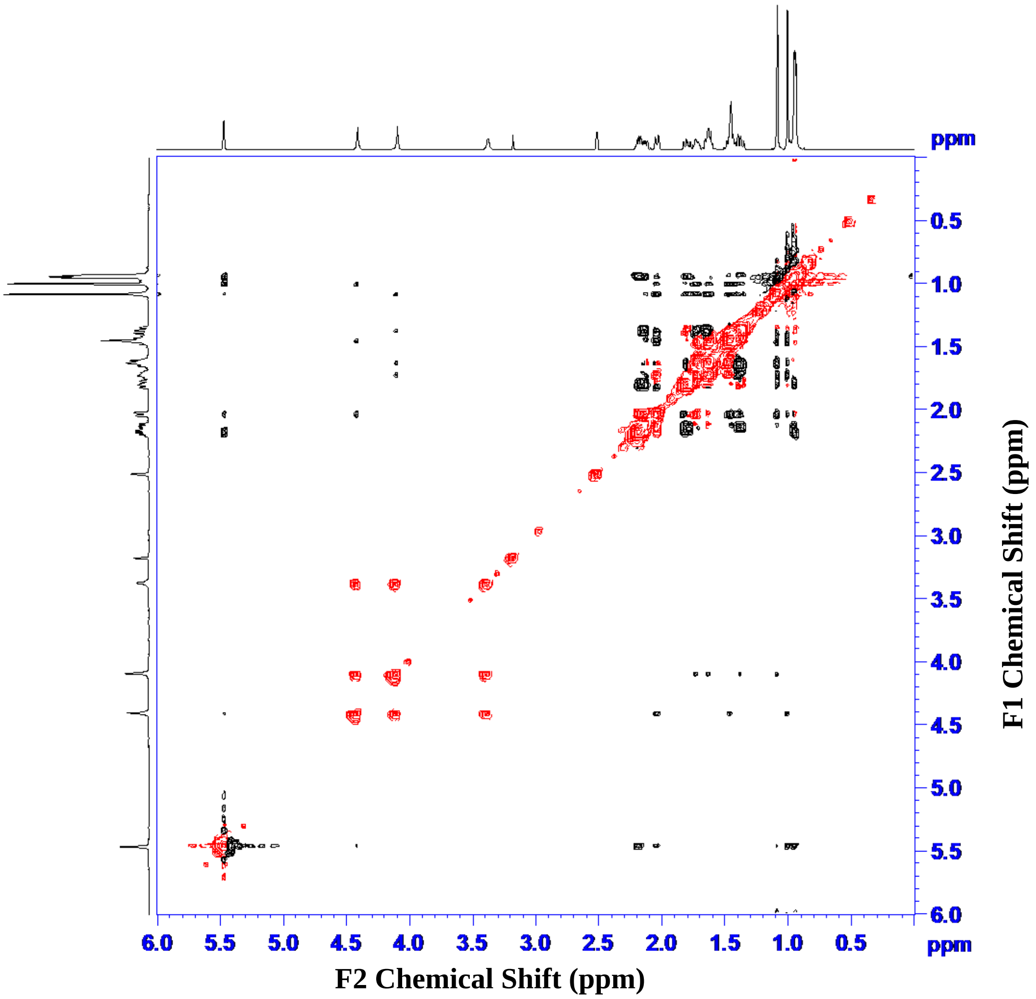

Figure S13. ^{1}H-NMR spectrum for compound **3**.

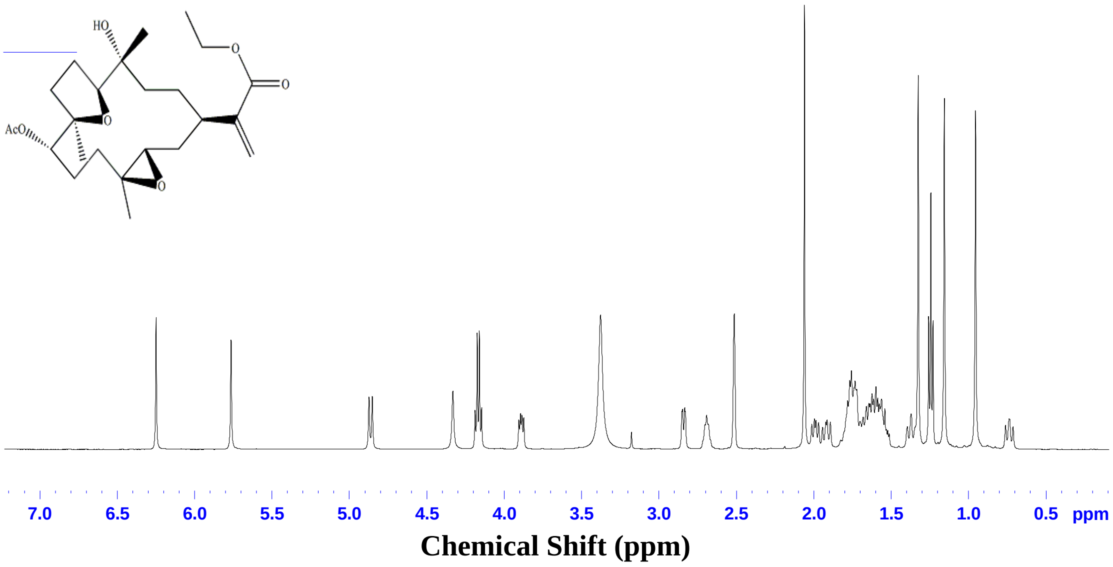

Figure S14. APT NMR spectrum for compound **3**.

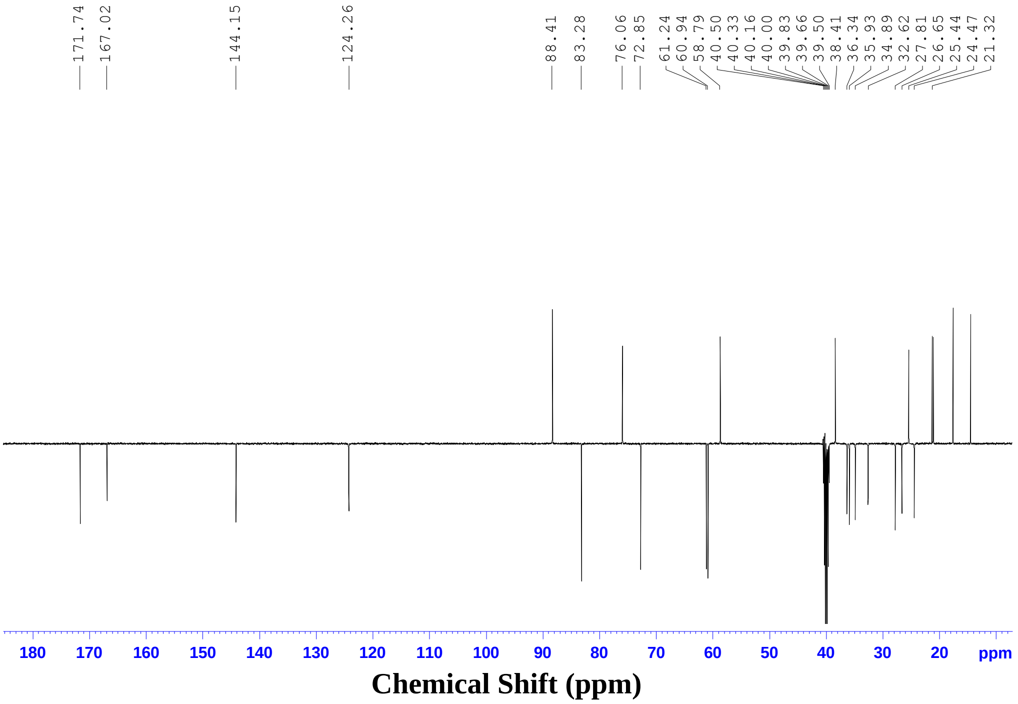

Figure S15. COSY NMR spectrum for compound **3**.

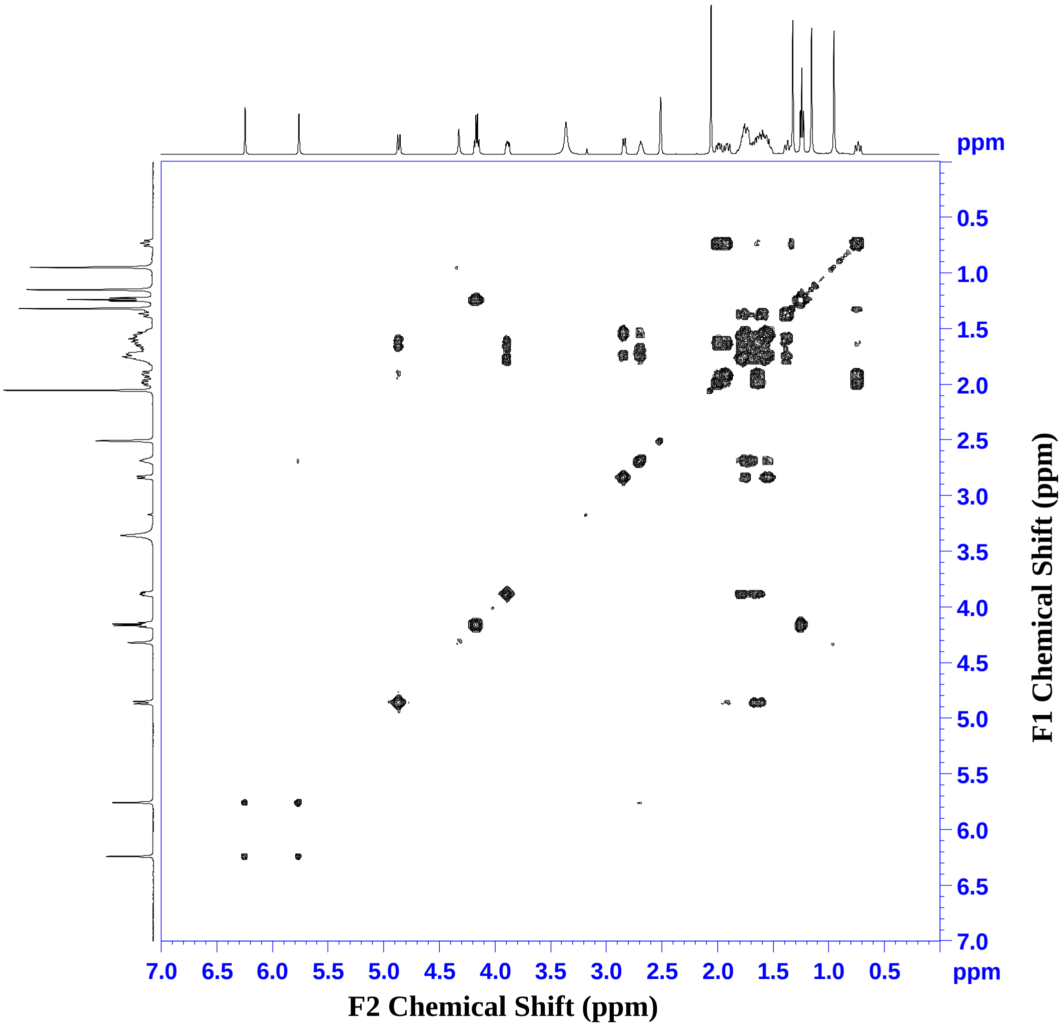

Figure S16. HSQC NMR spectrum for compound **3**.

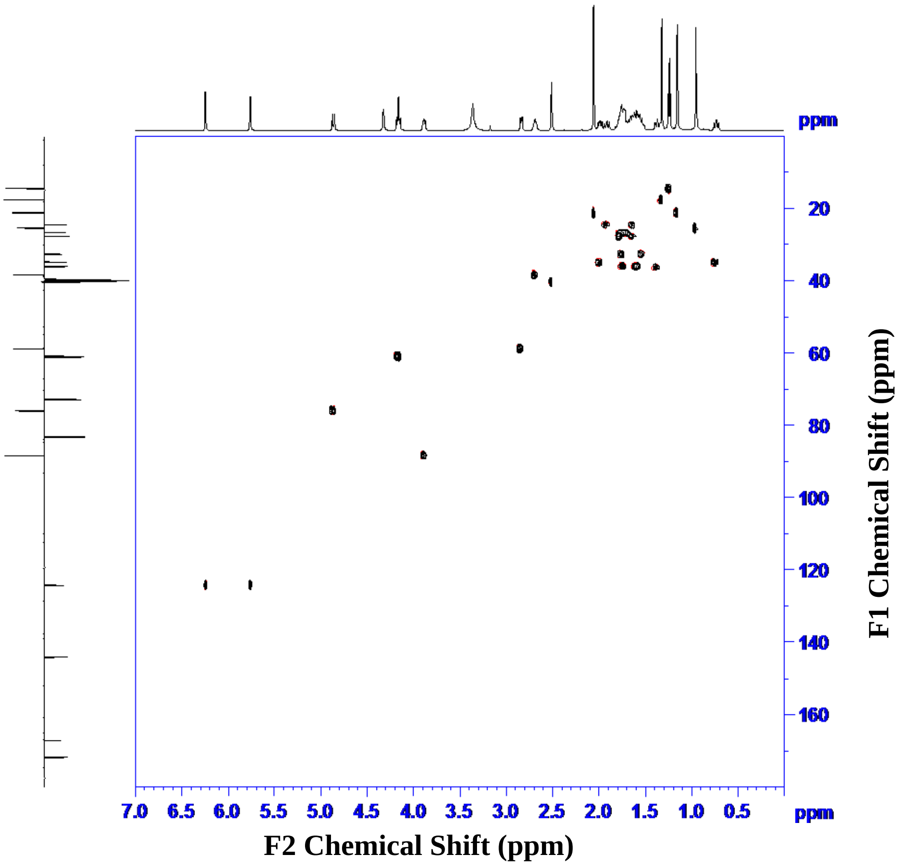

Figure S17. HMBC NMR spectrum for compound **3**.

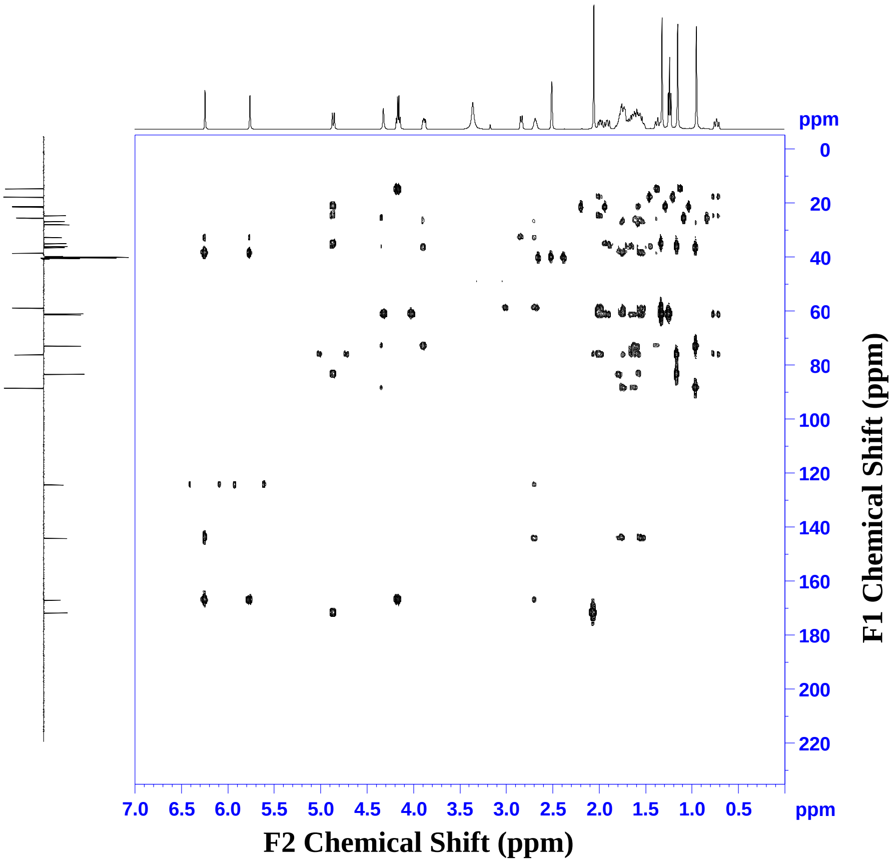

Figure S18. ROESY NMR spectrum for compound **3**.

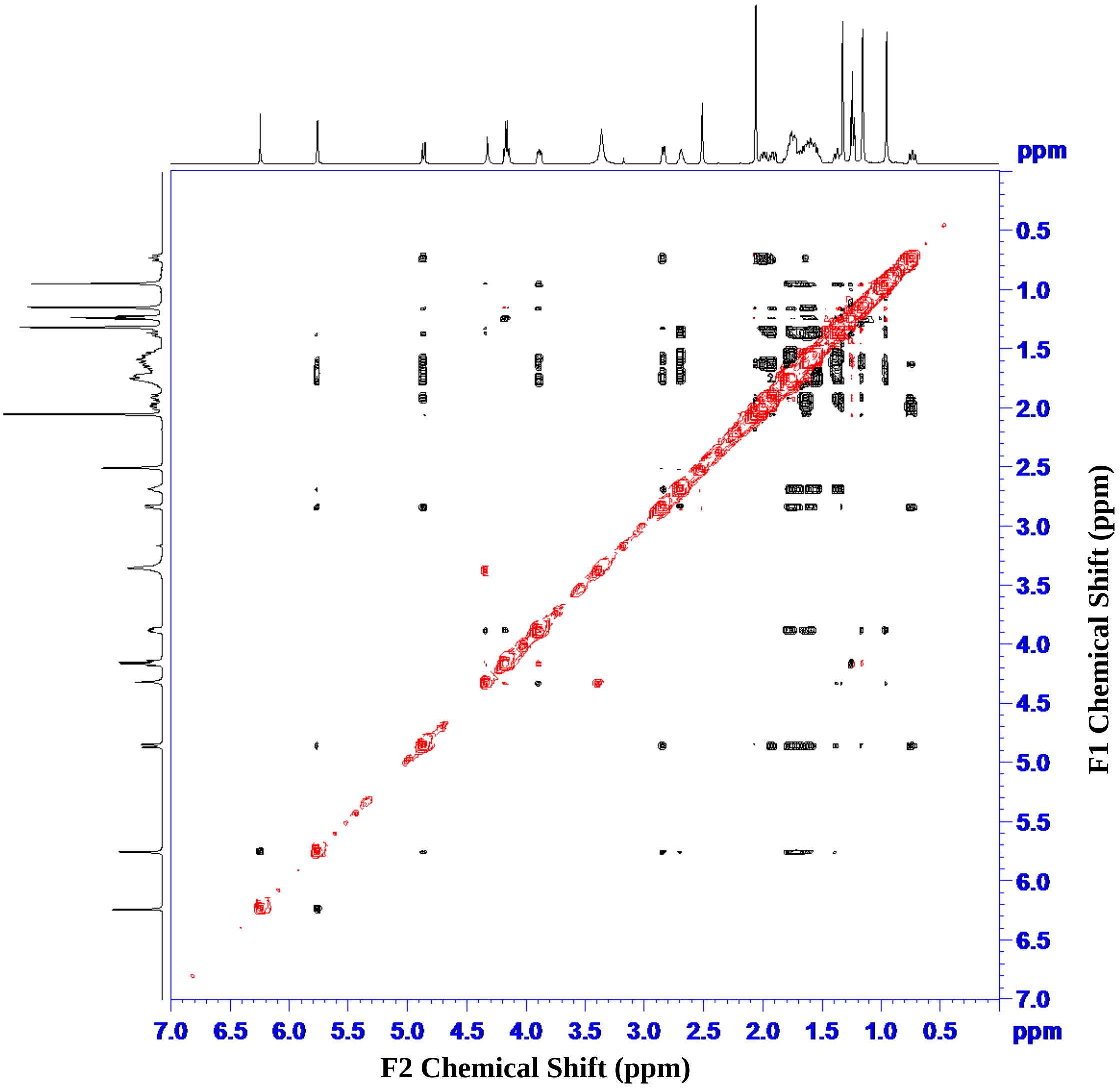

Figure S19. ^{1}H-NMR spectrum for compound **4**.

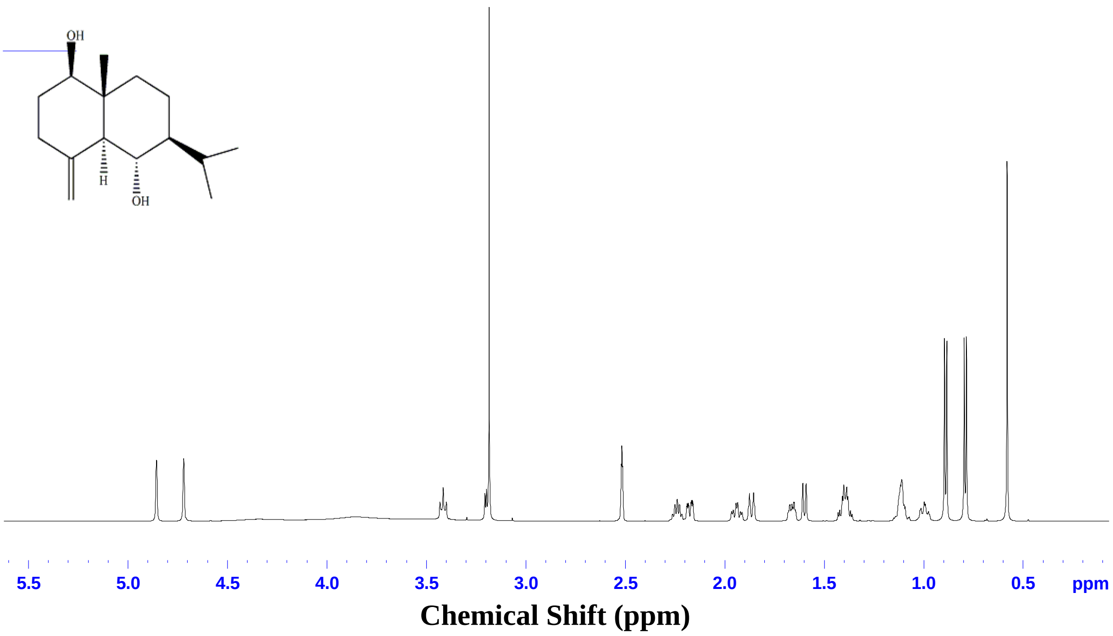

Figure S20. APT NMR spectrum for compound **4**.

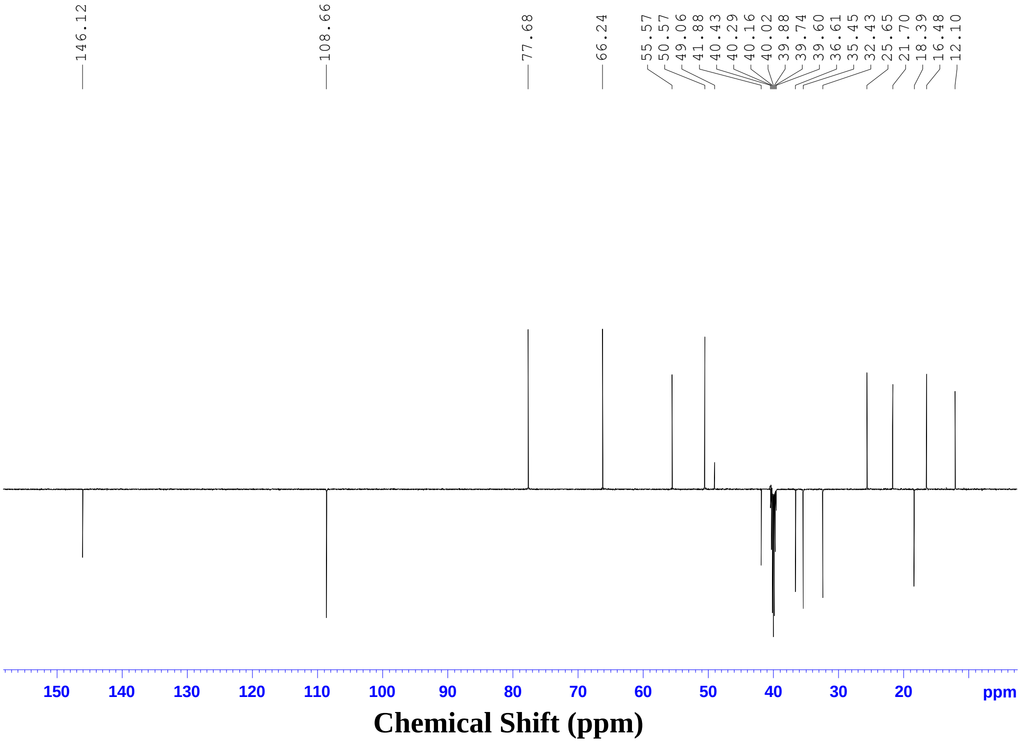

Figure S21. COSY NMR spectrum for compound **4**.

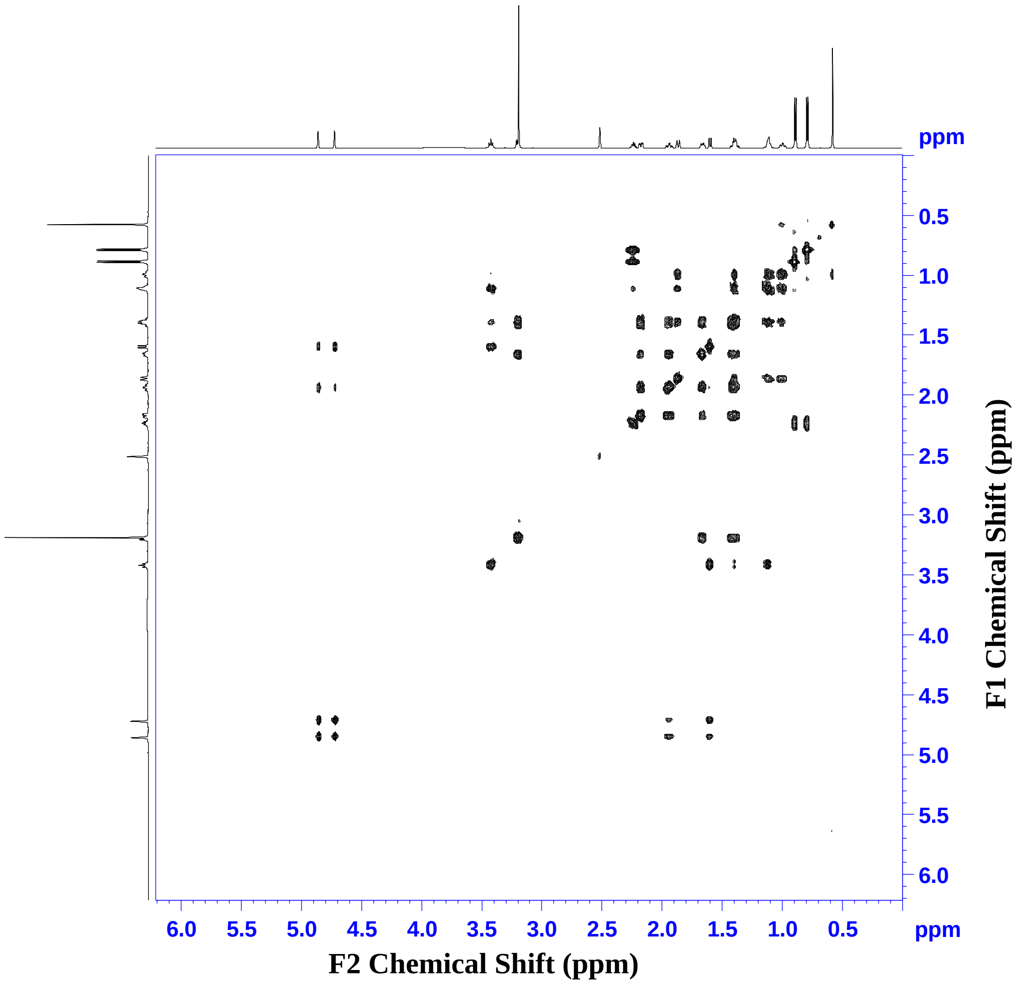

Figure S22. HSQC NMR spectrum for compound **4**.

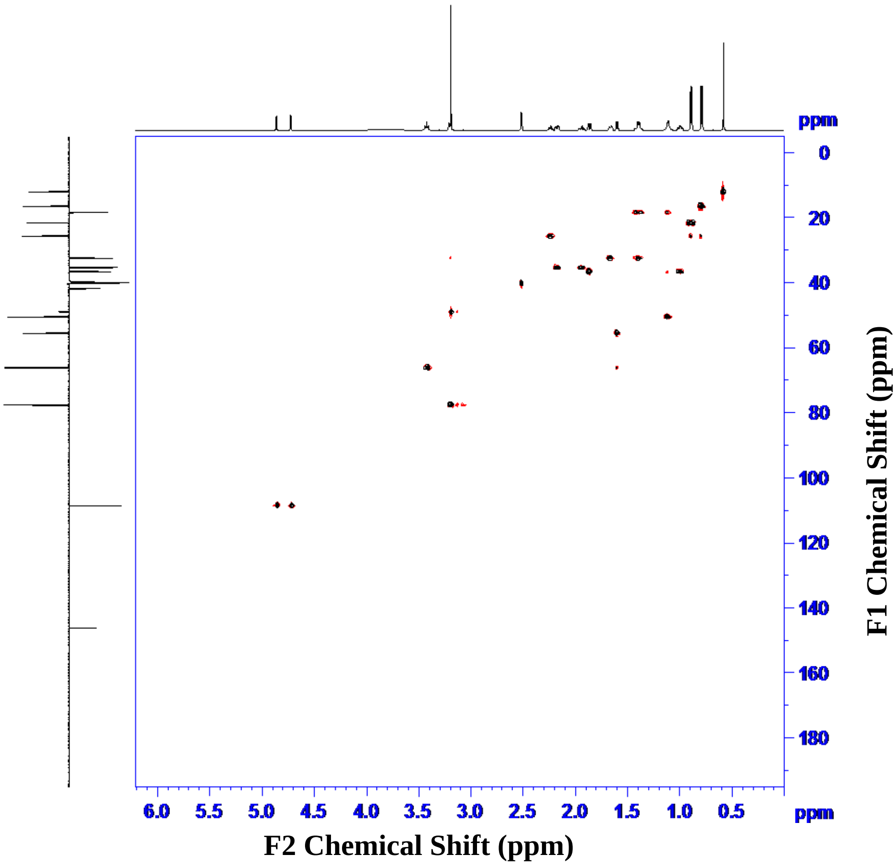

Figure S23. HMBC NMR spectrum for compound **4**.

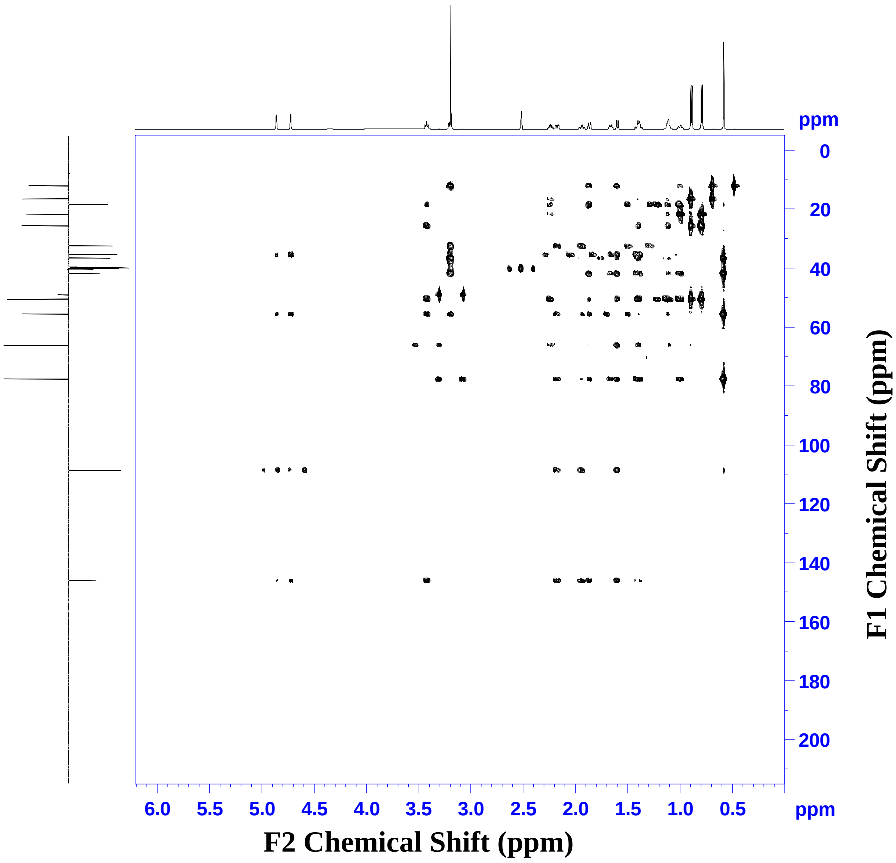

Figure S24. ROESY NMR spectrum for compound **4**.

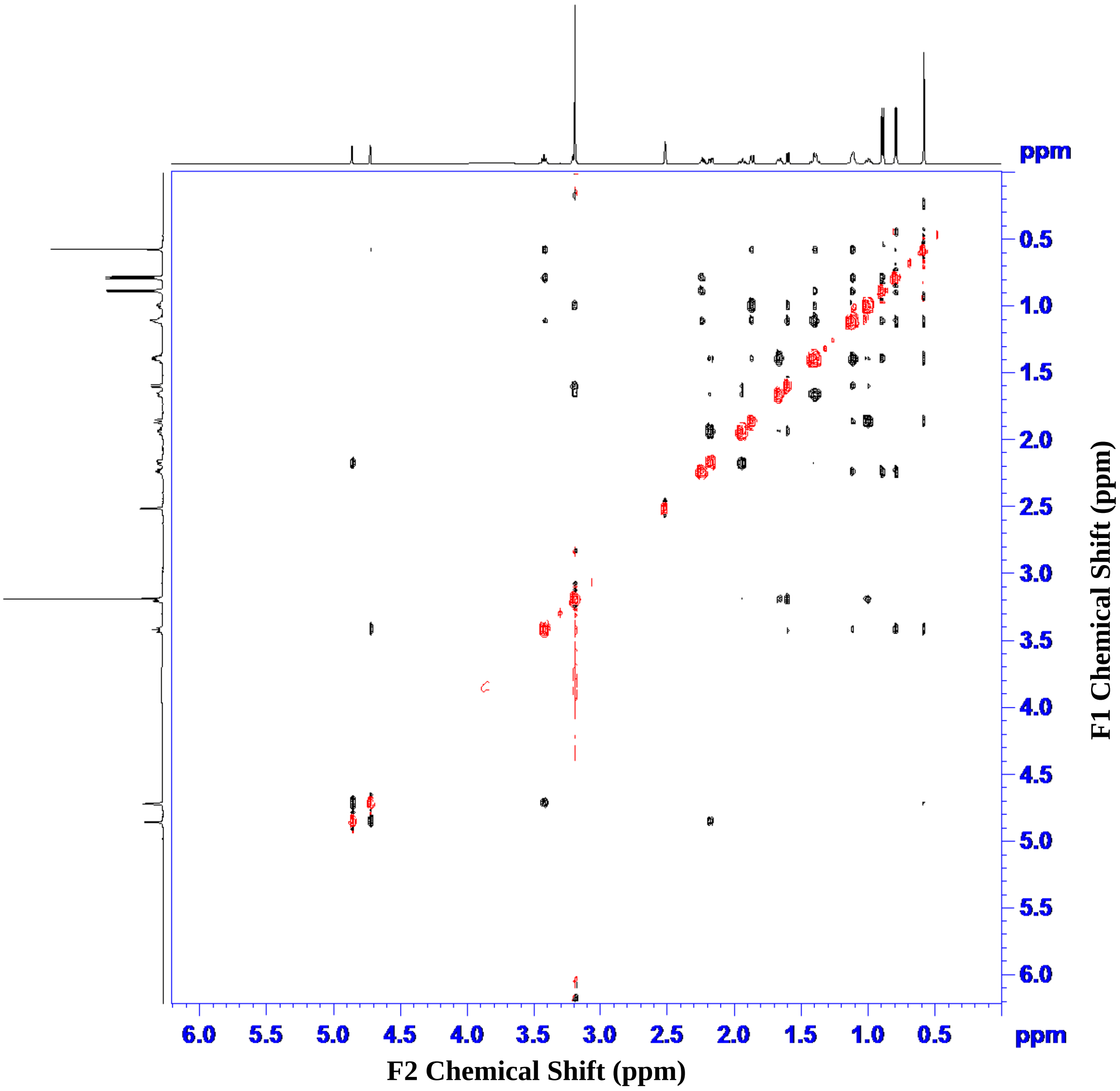

Figure S25. Validation result of PLS-DA for methanol extract samples by permutation.

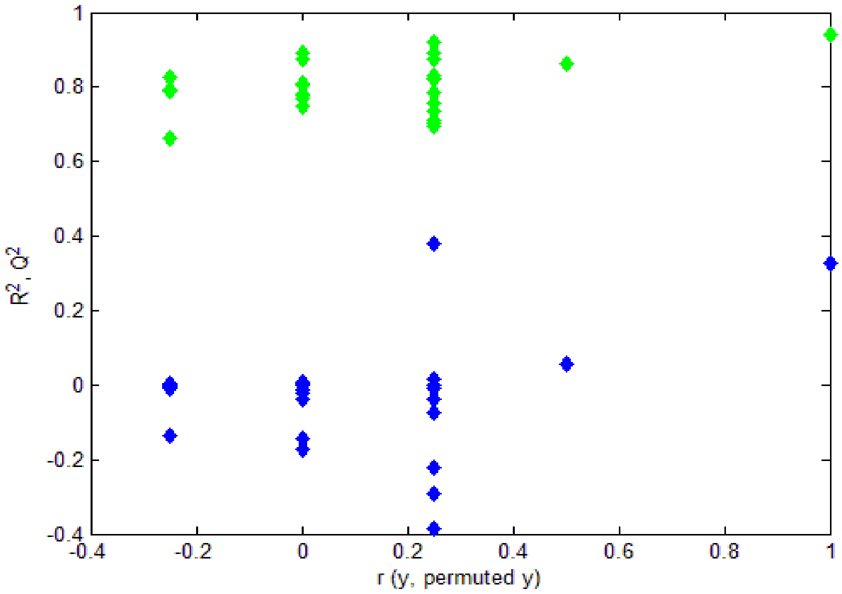

Figure S26. Validation result of PLS-DA for Fr.1 samples by permutation.

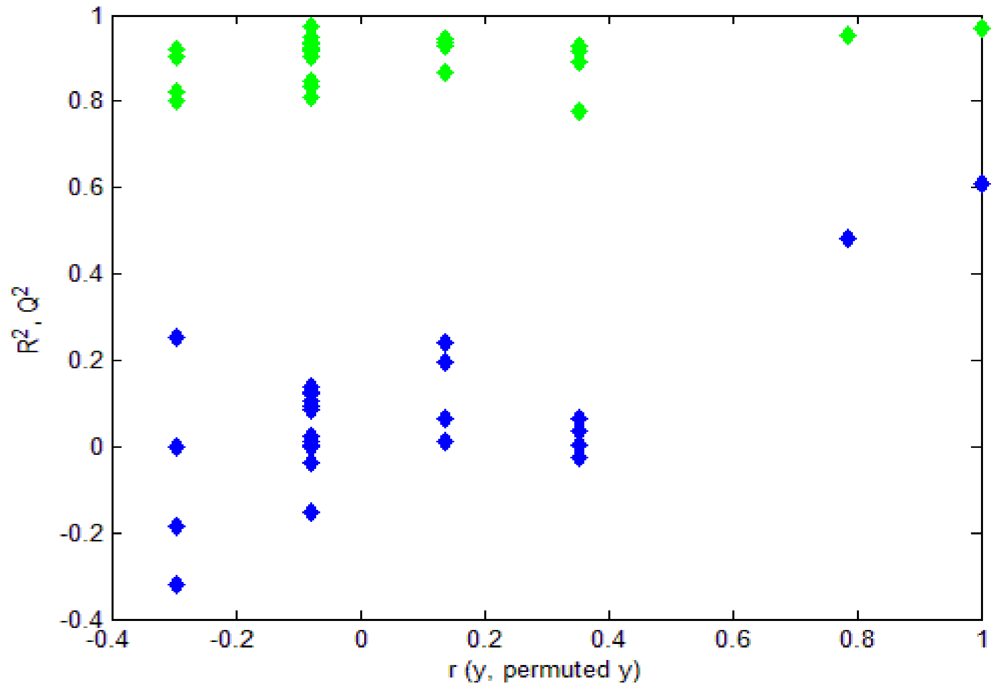

Figure S27. Validation result of PLS-DA for Fr.2 samples by permutation.

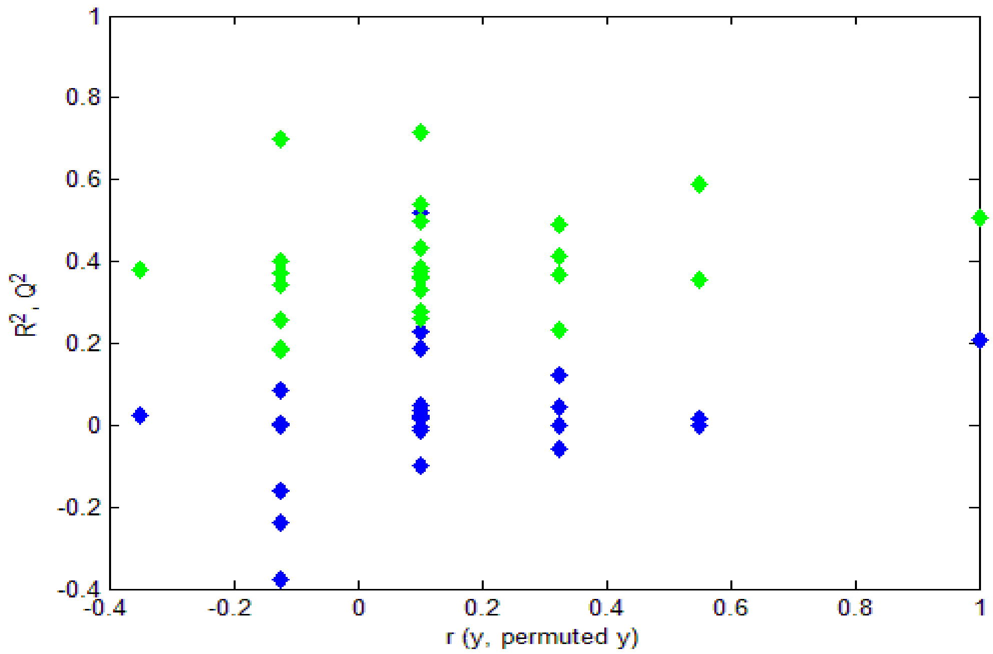

Figure S28. Validation result of PLS-DA for Fr.3 samples by permutation.

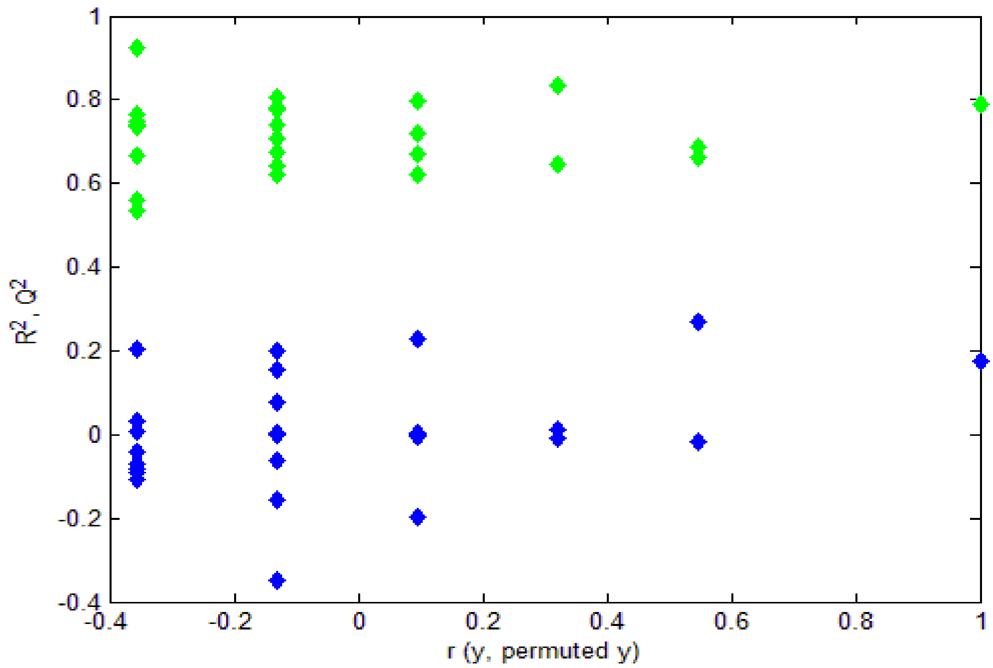

Figure S29. Validation result of PLS-DA for Fr.4 samples by permutation.

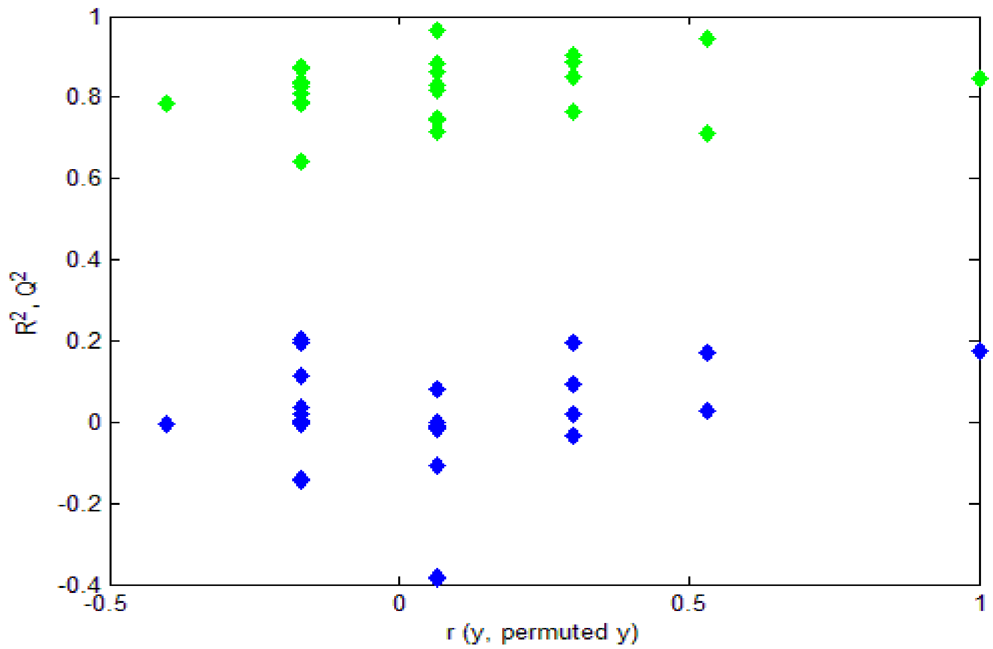

Figure S30. Validation result of PLS-DA for Fr.5 samples by permutation.

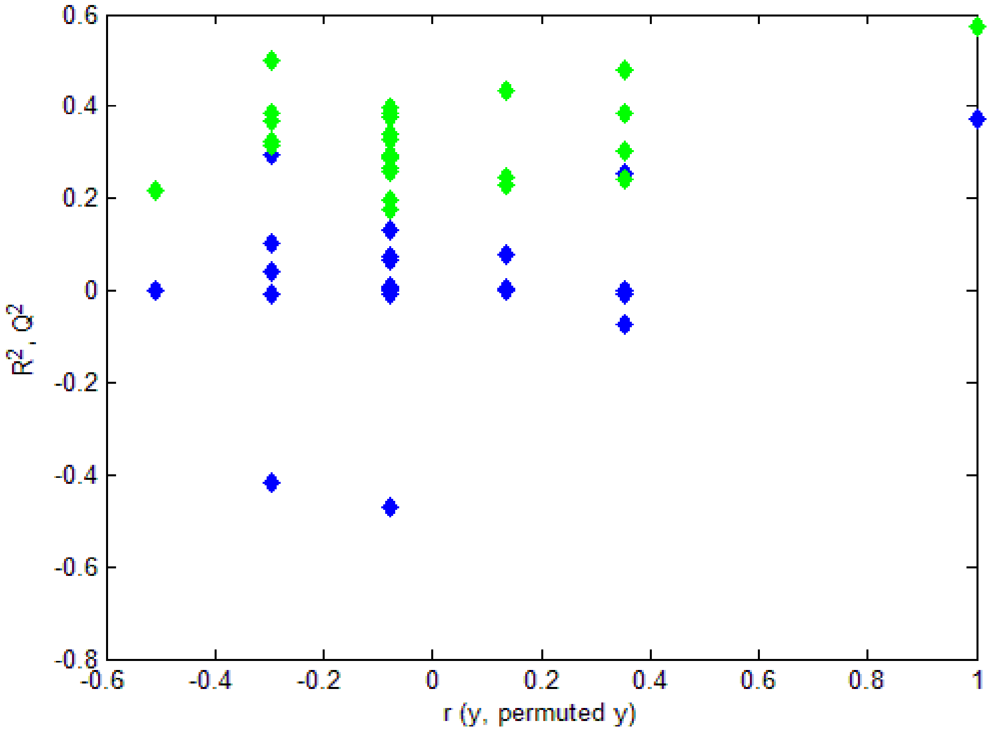

© 2014 by the authors; licensee MDPI, Basel, Switzerland. This article is an open access article distributed under the terms and conditions of the Creative Commons Attribution license (http://creativecommons.org/licenses/by/3.0/).
